# Home-Based Walking Exercise and Supervised Treadmill Exercise in Patients With Peripheral Artery Disease: An Individual Participant Data Meta-Analysis

**DOI:** 10.1001/jamanetworkopen.2023.34590

**Published:** 2023-09-21

**Authors:** Neela D. Thangada, Dongxue Zhang, Lu Tian, Lihui Zhao, W. Jack Rejeski, Karen J. Ho, Luigi Ferrucci, Bonnie Spring, Melina R. Kibbe, Tamar S. Polonsky, Michael H. Criqui, Mary M. McDermott

**Affiliations:** 1 Feinberg School of Medicine, Northwestern University, Chicago, Illinois; 2 Department of Health Research and Policy, Stanford University, Stanford, California; 3 Department of Health and Exercise Science, Wake Forest University, Winston-Salem, North Carolina; 4 Division of Intramural Research, National Institute on Aging, Bethesda, Maryland; 5 Department of Surgery, University of Virginia School of Medicine, Charlottesville; 6 Editor, *JAMA Surgery*; 7 Department of Medicine, Medical School, University of Chicago, Chicago, Illinois; 8 Department of Family Medicine and Public Health University of California San Diego, La Jolla; 9 Deputy Editor, *JAMA*

## Abstract

**Importance:**

Few people with lower extremity peripheral artery disease (PAD) participate in supervised treadmill exercise covered by the Center for Medicare and Medicaid Services. In people with PAD, the benefits of home-based walking exercise, relative to supervised exercise, remain unclear.

**Objective:**

To study whether home-based walking exercise improves 6-minute walk (6MW) more than supervised treadmill exercise in people with PAD (defined as Ankle Brachial Index ≤0.90).

**Data Sources:**

Data were combined from 5 randomized clinical trials of exercise therapy for PAD using individual participant data meta-analyses, published from 2009 to 2022.

**Study Selection:**

Of the 5 clinical trials, 3 clinical trials compared supervised treadmill exercise to nonexercise control (N = 370) and 2 clinical trials compared an effective home-based walking exercise intervention to nonexercise control (N = 349).

**Data Extraction and Synthesis:**

Individual participant-level data from 5 randomized clinical trials led by 1 investigative team were combined. The 5 randomized clinical trials included 3 clinical trials of supervised treadmill exercise and 2 effective home-based walking exercise interventions.

**Main Outcomes and Measures:**

Change in 6MW distance, maximum treadmill walking distance, and Walking Impairment Questionnaire at 6-month follow-up. The supervised treadmill exercise intervention consisted of treadmill exercise in the presence of an exercise physiologist, conducted 3 days weekly for up to 50 minutes per session. Home-based walking exercise consisted of a behavioral intervention in which a coach helped participants walk for exercise in or around home for up to 5 days per week for 50 minutes per session.

**Results:**

A total of 719 participants with PAD (mean [SD] age, 68.8 [9.5] years; 46.5% female) were included (349 in a home-based exercise clinical trial and 370 in a supervised exercise trial). Compared with nonexercise control, supervised treadmill exercise was associated with significantly improved 6MW by 32.9 m (95% CI, 20.6-45.6; *P* < .001) and home-based walking exercise was associated with significantly improved 6MW by 50.7 m (95% CI, 34.8-66.7; *P* < .001). Compared with supervised treadmill exercise, home-based walking exercise was associated with significantly greater improvement in 6MW distance (between-group difference: 23.8 m [95% CI, 3.6, 44.0; *P* = .02]) but significantly less improvement in maximum treadmill walking distance (between-group difference:-132.5 m [95% CI, −192.9 to −72.1; *P* < .001]).

**Conclusions and Relevance:**

In this individual participant data meta-analyses, compared with supervised exercise, home-based walking exercise was associated with greater improvement in 6MW in people with PAD. These findings support home-based walking exercise as a first-line therapy for walking limitations in PAD.

## Key Points

**Question:**

Is home-based walking exercise associated with greater improvement in 6-minute walk (6MW) distance compared with supervised treadmill exercise?

**Findings:**

In this individual participant data meta-analysis that included 719 participants from 5 randomized clinical trials of exercise for peripheral artery disease (PAD), compared with supervised exercise, home-based walking exercise was associated with significantly greater improvement in 6MW distance (23.8 m), but significantly less improvement in maximal treadmill walking distance.

**Meaning:**

The findings suggest that compared with supervised treadmill exercise, home-based walking exercise was associated with greater improvement in 6MW distance but not treadmill walking distance, in people with PAD.

## Teaser

This individual participant data meta-analysis compares 6-minute walk test distance in individuals with peripheral artery disease who received either home-based walking exercise or supervised treadmill.

## Introduction

Supervised treadmill exercise is first-line therapy for walking impairment among people with lower extremity peripheral artery disease (PAD).^1,2^ However, traveling to a center for supervised exercise is burdensome and most people with PAD do not participate.^2,3,4^ Home-based walking exercise, consisting of walking exercise conducted in or near the home without physical presence of a coach, is more convenient and less burdensome.^3^ However, uncertainty exists regarding the benefits of home-based walking exercise for PAD.^3^ Furthermore, supervised treadmill exercise has been considered effective based on its effects on treadmill walking, but the 6-minute walk (6MW) distance is more relevant to walking in daily life than treadmill walking.^5^ To our knowledge, only 1 clinical trial has directly compared the effects of home-based walking exercise with supervised treadmill exercise on 6MW distance in people with PAD.^6^

This study combined data from randomized clinical trials that compared supervised treadmill exercise to a control group with data from clinical trials that compared effective home-based walking exercise to a control group to compare effect sizes of home-based walking exercise interventions with effect sizes of supervised treadmill exercise on outcomes of 6MW, treadmill walking distance, and patient-reported outcome measures in people with PAD. The study aims were as follows: first, to compare changes in study outcomes between home-based walking exercise and the control group and between supervised treadmill exercise and the control group; second, to combine data from the home-based exercise and supervised exercise trials and use an individual participant level analysis to more directly compare home-based walking exercise and supervised treadmill exercise. We hypothesized that in people with PAD, home-based walking exercise would be associated with greater improvement in 6MW distance and patient reported outcomes, compared with supervised treadmill exercise and that supervised treadmill exercise would be associated with greater improvement in treadmill walking distance, compared with home-based exercise.

## Methods

Five randomized clinical trials published from 2009 to 2022 from a single laboratory that compared either supervised treadmill or effective home-based walking exercise with a nonexercise control group in people with PAD were included.^7,8,9,10,11^ These clinical trials were selected because they all measured 6MW distance, treadmill walking performance, and the Walking Impairment Questionnaire (WIQ) at baseline and 6-month follow-up. The supervised exercise trials used the same supervised exercise intervention and the home-based walking exercise clinical trials used similar behavioral methods to help participants adhere to home-based exercise. Home-based walking exercise programs that demonstrated significant benefit were eligible for inclusion, since these exercise interventions, but not ineffective home-based exercise interventions, could potentially be incorporated into clinical practice. One home-based walking exercise intervention was not included because the trial did not include 6MW distance and treadmill testing at baseline and 6-month follow-up and because the exercise intervention was not effective and therefore not appropriate for implementation into clinical practice.^12^ The 5 randomized clinical trials were approved by the institutional review boards at participating sites. All participants provided written informed consent.

The clinical trials of supervised treadmill exercise were the Study in Leg Circulation (SILC), Progenitor Cell Release Plus Exercise to Improve Functional Performance in PAD (PROPEL), and Effect of Telmisartan on Walking Performance in Patients with Lower Extremity PAD (TELEX).^7,8,9^ Clinical trials of home-based walking exercise were Group Oriented Arterial Leg Study (GOALS) and Effect of Low-Intensity vs High-Intensity Home-Based Walking Exercise on Walk Distance in Patients with PAD (LITE).^10,11^ The SILC trial compared strength training and supervised treadmill exercise training, respectively, to a control group. Since the focus of these analyses was walking exercise, the strength training group was excluded from analyses. The PROPEL trial studied supervised exercise with and without granulocyte macrophage colony stimulating factor (GM-CSF). The TELEX trial studied supervised treadmill exercise with and without telmisartan. Because neither GM-CSF nor telmisartan significantly improved 6MW distance and there were no statistically significant interactions of either drug on the effects of exercise on walking performance, participants randomized to drug therapies were included in analyses.^8,9^ The LITE trial studied home-based exercise at a pace inducing ischemic leg symptoms (high-intensity) or at a comfortable pace without ischemic symptoms (low-intensity). Because low-intensity exercise was ineffective, only participants in the high-intensity group were included in analyses.

### Participants

All clinical trials were led by Northwestern University in Chicago, IL. Additional sites were New Orleans, Louisiana, (LITE and TELEX), Pittsburgh, Pennsylvania (LITE), and Minneapolis, Minnesota (LITE). Participants were identified from patients with PAD at each medical center and from advertisements using newspapers, radio, or public buses and trains. Recruitment postcards were mailed to people aged 50 and older living in communities near recruitment sites.

Comorbidities and leg symptoms were ascertained by questionnaire.^7,8,9,10,11^ Race data were collected using an open-ended question and responses were recorded in fixed categories. This information was required by the National Institutes of Health (National Heart, Lung, and Blood Institute) and helped assess generalizability of results. Height and weight were measured at baseline. Body mass index was calculated as kilograms per meters squared.

#### Inclusion Criteria

The inclusion criterion was an ankle brachial index (ABI) of 0.90 or less for PROPEL, TELEX, GOALS and LITE and ABI≤0.95 for SILC. Participants with ABI greater than 0.90 and radiographic or vascular laboratory documenting lower extremity atherosclerosis causing 70% stenosis or more in lower extremity arteries were eligible. Individuals with an ABI of 0.91 to 1.00 at baseline whose ABI dropped by 20% or more after a heel-rise test were eligible.^13^

#### Exclusion Criteria

Potential participants with foot ulcers, chronic limb threatening ischemia, significant visual or hearing impairment, walking impairment not due to PAD, above or below-the-knee amputations, and those wheelchair bound were excluded. People with recent or planned major surgery or revascularization and those who had participated in cardiac rehabilitation or another supervised exercise program within 3 months before enrollment were excluded. People with abnormal exercise stress tests at baseline were excluded unless subsequent cardiac testing showed no significant coronary ischemia.

#### Ankle-Brachial Index

Systolic blood pressures in bilateral brachial, dorsalis pedis, and posterior tibial arteries were measured using a handheld Doppler probe (Nicolet Vascular Pocket Dop II; Nicolet Biomedical Inc) as previously described.^7,8,9,10,11^

### Randomization and Interventions

The randomly permuted block method was used for randomization for all clinical trials (eTable 1 in Supplement 1).^7,8,9,10,11^

#### Supervised Treadmill Exercise Intervention

Supervised treadmill exercise interventions consisted of 3 exercise sessions per week with an exercise physiologist at an exercise center. Participants were asked to walk for exercise at a pace inducing ischemic leg symptoms for 10 to 15 minutes in week 1, working up to 50 minutes of exercise per session if possible.^7,8,9^ The program was individualized according to each participant’s ability. Treadmill speed and grade were increased such that participants experienced significant ischemic leg symptoms within 10 minutes of each exercise bout. Walking exercise minutes were recorded but not entered into the study database.

#### Home-Based Walking Exercise Intervention

In both home-based walking exercise interventions, participants were asked to walk for exercise near or around home, 5 days per week beginning at 15-20 minutes per day (excluding rest), working up to a maximum of 50 minutes of exercise per session, if possible.^10,11^ Walking exercise interventions were individualized according to each participant’s ability.

The GOALS intervention was a Group Mediated Cognitive Behavioral (GMCB) intervention in which participants met weekly at the exercise facility with other participants and the coach.^10^ In LITE, participants were randomized to either walking exercise at a pace inducing ischemic leg symptoms (high-intensity), walking exercise without ischemic leg symptoms (low-intensity), or control.^11^ The LITE intervention began with 4 weekly in-person individual visits with the coach and subsequently consisted of weekly telephone calls from the coach. LITE participants randomized to exercise wore an accelerometer (ActiGraph; ActiGraph LLC) during exercise to record exercise intensity and minutes of exercise each day. Accelerometer data were uploaded on a website and viewed by the participant and coach. Both GOALS and LITE incorporated behavioral changes methods including goal setting, self-monitoring, building self-efficacy, and coach feedback.

#### Control Groups

In the supervised exercise trials, the control group attended scheduled meetings at the medical center with other participants for 1-hour educational sessions. Topics included immunizations, cancer screening, and others, but did not cover exercise.^7,8,9^ In GOALS, participants met weekly for lectures on similar topics.^10^ In LITE, participants had weekly telephone calls on topics related to healthy aging.^11^

#### 6-Minute Walk

Using scripted instructions, participants were asked to walk up and down a 100-foot hallway for 6 minutes with the goal of walking as much distance as possible in 6 minutes.^5^ In PAD, 8 m represents a small clinically important difference, and 20 m represents a large clinically important difference.^14,15^

#### Treadmill Walking Performance

The Gardner-Skinner treadmill protocol was used to measure maximal treadmill walking distance and pain-free walking distance using established methods.^16^

#### Walking Impairment Questionnaire

The Walking Impairment Questionnaire (WIQ) is a patient-reported PAD specific questionnaire with 3 domains (distance, walking speed, and stair-climbing) scored on a 0 to 100 scale (100-best).^17^ The distance score measures patient reported difficulty walking specific distances ranging from across the room up to 1500 feet. The speed score measures reported difficulty walking speeds ranging from a slow walk to a jog. The stair-climbing score measures difficulty climbing 1 to 3 stair flights.

### Statistical Analyses

Individuals who participated in more than 1 clinical trial were included only for the first clinical trial in which they participated. Participants were analyzed in the group to which they were randomized, regardless of intervention adherence. Data from the 3 supervised treadmill exercise clinical trials were combined and data from the 2 home-based exercise trials were combined. Baseline characteristics were reported for the supervised exercise group, home-based exercise group, and each (supervised or home-based) control group as median (IQR) for continuous variables and No. (%) for categorical variables.

The analysis of covariance was performed for each study to investigate study-specific between-group differences in each outcome between participants randomized to the exercise group and the corresponding control group. An individual participant data meta-analysis was performed to identify between-group differences in each outcome between participants randomized to supervised exercise or home-based exercise. The individual participant data meta-analysis was conducted using analysis of covariance assuming a common between-group difference across studies, adjusting for age, sex, race, baseline value for each outcome, study, and baseline variables that differed significantly between participants in supervised and home-based exercise groups at *P* < .05 (cigarette smoking, history of myocardial infarction, and heart failure). Testing was 2-sided. A *P* < .05 was considered statistically significant. SAS version 9.4 (SAS Institute) was used for analyses.

## Results

Of 719 unique participants, 370 participated in a supervised exercise trial and 349 in a home-based exercise trial (Table 1). Ninety-two people participated in more than 1 clinical trial and were included only in the first trial they participated in. Overall, mean (SD) age was 68.6 (9.5 years), 334 (46.5%) were women, 2 (0.3%) were American Indian/Alaska Native, 9 (1.3%) were Asian, 415 (57.7%) were Black, 2 (0.3%) were Native Hawaiian/Pacific Islander, 282 (39.2%) were White, and 5 (0.7%) were other/unknow/not reported. Among participants in a home-based exercise trial, those randomized to exercise had a higher prevalence of prior myocardial infarction at baseline compared with control. Among participants in a supervised exercise trial, those randomized to exercise had a lower prevalence of heart failure compared with control. Among those randomized in the exercise group in the LITE trial, mean (SD) exercise minutes per week was 74.98 (64.1).

**Table 1.** Baseline Characteristics of Participants

Baseline variable	Supervised treadmill exercise trials		Home-based walking exercise trials		*P* value	
	Supervised exercise (n = 182)	Attention control (n = 188)	Home-based exercise (n = 198)	Attention control (n = 151)	Control^a^	Exercise^b^
Age, median (IQR), y	67.50 (62.00-75.00)	66.00 (60.00-73.00)	69.00 (63.00-74.00)	71.00 (64.00-77.00)	<.001	.36
Sex, No. (%)						
Female	80 (43.96)	79 (42.02)	99 (50.00)	76 (50.33)	.13	.24
Male	102 (56.04)	109 (57.98)	99 (50.00)	75 (49.67)		
Race, No. (%)^c^						
American Indian/Alaska Native	0	1 (0.53)	1 (0.51)	0	<.001	.70
Asian	3 (1.65)	0	1 (0.51)	5 (3.31)		
Black	104 (57.14)	124 (65.96)	116 (58.59)	71 (47.02)		
Native Hawaiian/Other Pacific Islander	0	2 (1.06)	0	0		
More than 1 race	2 (1.10)	1 (0.53)	1 (0.51)	0		
White	71 (39.01)	58 (30.85)	78 (39.39)	75 (49.67)		
Other/Unknown/not reported	2 (1.10)	2 (1.06)	1 (0.51)	0		
ABI, median (IQR)	0.65 (0.54-0.83)	0.67 (0.53-0.81)	0.68 (0.58-0.81)	0.73 (0.56-0.85)	.06	.21
BMI, median (IQR)	29.79 (25.66-33.77)	29.28 (25.08-33.82)	28.40 (24.78-34.58)	28.44 (24.64-33.88)	.38	.47
Current smoker, No. (%)	68 (37.36)	68 (36.17)	45 (22.73)	32 (21.19)	.003	.002
Myocardial infarction, No. (%)	33 (18.23)	39 (20.74)	41 (20.71)	19 (12.58)	.047	.54
Heart failure, No. (%)	13 (7.22)	28 (14.89)	28 (14.14)	17 (11.26)	.33	.03
Stroke, No. (%)	32 (17.68)	32 (17.02)	34 (17.17)	32 (21.19)	.33	.90
Angina, No. (%)	26 (14.29)	24 (12.83)	34 (17.17)	25 (16.67)	.32	.44
Pulmonary disease, No. (%)	24 (13.19)	30 (16.13)	30 (15.15)	22 (14.57)	.69	.58
Cancer, No. (%)	37 (20.33)	30 (15.96)	34 (17.17)	30 (20.00)	.33	.43
Diabetes, No. (%)	69 (37.91)	71 (37.77)	72 (36.36)	64 (42.38)	.39	.75
IC, No. (%)	53 (29.12)	48 (25.53)	49 (24.75)	31 (20.53)	.23	.62
Leg pain not intermittent claudication, No. (%)	117 (64.29)	133 (70.74)	136 (68.69)	109 (72.19)		
Asymptomatic (no exertional leg pain), No. (%)	12 (6.59)	7 (3.72)	13 (6.57)	11 (7.28)		
6MW distance, median (IQR), m	326.29 (262.13-396.85)	339.55 (274.32-410.26)	349.15 (265.79-405.69)	348.08 (275.23-396.24)	.66	.25
Total treadmill distance, median (IQR), m	294.15 (169.87-450.62)	325.00 (198.49-507.84)	327.68 (168.98-542.71)	335.28 (212.79-497.11)	.77	.24
Treadmill distance at onset of leg symptom median (IQR), m	120.25 (65.27-231.57)	120.25 (80.91-238.72)	116.68 (78.68-192.23)	116.23 (67.06-221.73)	.42	.69
WIQ distance score, median (IQR)	25.36 (10.16-44.60)	28.66 (11.08-50.36)	27.56 (10.01-50.00)	28.91 (12.14-48.86)	.99	.39
WIQ speed score, median (IQR)	25.00 (14.13-50.00)	28.26 (15.22-50.00)	29.35 (15.22-50.00)	32.61 (18.48-50.00)	.35	.51
WIQ stair-climbing score, median (IQR)	41.67 (25.00-66.67)	41.67 (25.00-66.67)	41.67 (25.00-66.67)	41.67 (29.17-66.67)	.84	.46

Abbreviations: 6MW, 6-minute walk; ABI, Ankle Brachial Index; BMI, body mass index (calculated as weight in kilograms divided by height in meters squared); IC, intermittent claudication; WIQ, Walking Impairment Questionnaire (range, 0-100, with 100-indicating best).

^a^ *P* value comparing exercise group from supervised treadmill trials vs attention control home-based exercise trials.

^b^ *P* value comparing attention control from supervised treadmill trials vs exercise group from home-based exercise trials.

^c^ Race data were collected using an open-ended question and responses were recorded in fixed categories.

### Supervised Treadmill Exercise Compared With Control

**Figure 1.**
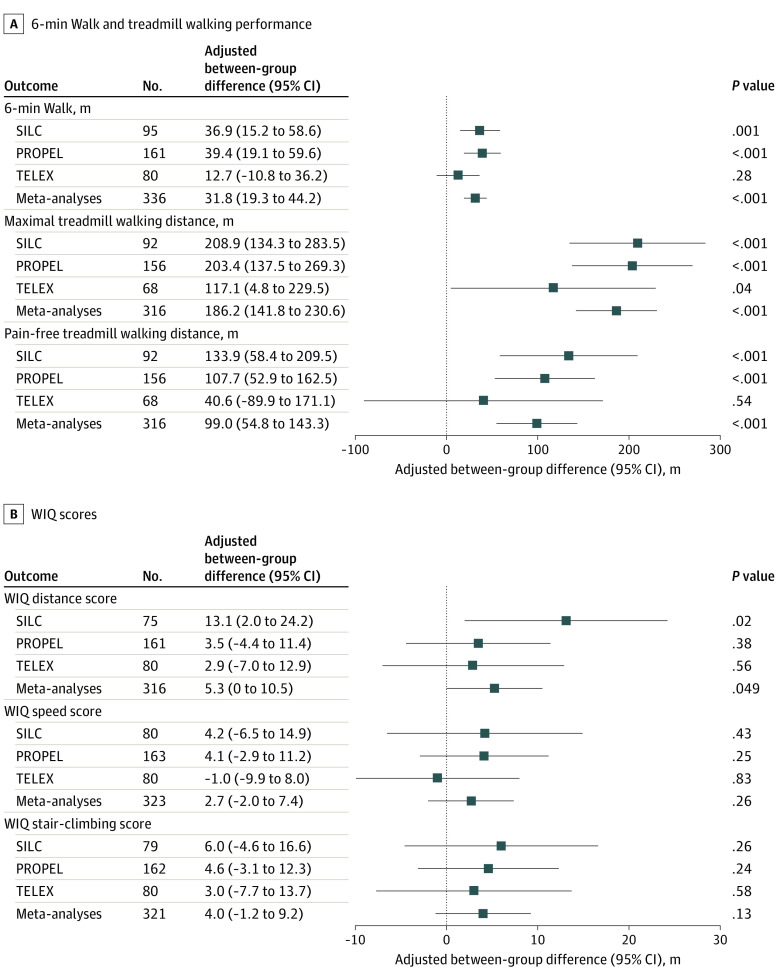
Adjusted Results From Meta-Analysis of the Supervised Treadmill Exercise Randomized Clinical Trials A, 6-minute Walk and treadmill outcomes; B, Walking Impairment Questionnaire (WIQ) outcomes for clinical trials of supervised treadmill exercise compared with control. Individual participant data meta-analyses were performed using analysis of covariance adjusting for study, age, sex, race, smoking, myocardial infarction, heart failure, and the baseline measure of outcome of interest to estimate the adjusted between-group difference in outcomes among those randomized to supervised treadmill exercise and to nonexercise control.

Compared with control, supervised treadmill exercise was associated with significantly improved mean (SD) 6MW distance (26.6 [59.5] vs −6.19 [59.1] m; adjusted between-group difference: 31.8 m [95% CI, 19.3-44.2]; *P* < .001) and mean (SD) maximum treadmill walking distance (235.7 [220.3] vs 48.22 [175.4] m; adjusted between-group difference: 186.2 m [95% CI, 141.77-230.55]; *P* < .001) (Figure 1; eTable 2 in Supplement 1). Compared with control, supervised treadmill exercise was associated with significantly improved mean (SD) WIQ distance scores (10.1 [25.3] vs 4.4 [23.7]; adjusted between-group difference: 5.3 [95% CI, 0.03-10.5]; *P* = .049) but not with WIQ stair-climbing or speed scores.

### Home-Based Walking Exercise Compared With Control

**Figure 2.**
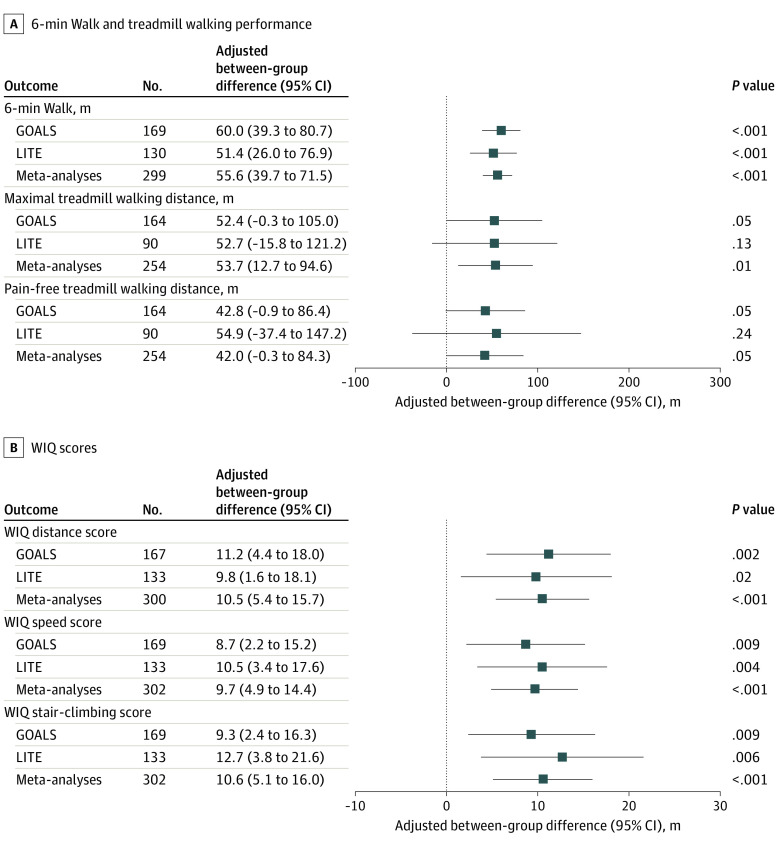
Adjusted Results From Meta-Analysis of Home-Based Walking Exercise Randomized Clinical Trials A, 6-minute Walk and treadmill outcomes; B, Walking Impairment Questionnaire (WIQ) outcomes for clinical trials of home-based walking exercise compared with control. Individual participant data meta-analyses were performed using analysis of covariance adjusting for study, age, sex, race, smoking, myocardial infarction, heart failure, and the baseline measure of outcome of interest to estimate the adjusted between-group differences in outcomes among those randomized to home-based walking exercise and to nonexercise control.

Compared with control, home-based walking exercise was associated with significantly improved mean (SD) 6MW distance (38.6 [72.1] vs −11.4 [63.9] m; adjusted between-group difference: 55.6 m [95% CI, 39.7-71.5]; *P* < .001) and mean (SD) maximum treadmill walking distance (92.8 [167.9] vs 34.6 [151.0] m; adjusted between-group difference: 53.7 m [95% CI, 12.7-94.6]; *P* = .011) (Figure 2; eTable 3 in Supplement 1). Compared with control, those randomized to home-based exercise had significantly greater improvement in mean (SD) WIQ distance score (10.2 [24.4] vs 1.34 [21.6]; adjusted between-group difference: 10.50 [95% CI, 5.4-15.7]; *P* < .001), mean (SD) WIQ speed score (9.32 [22.4] vs −0.58 [23.7]; adjusted between-group difference: 9.67 [95% CI, 4.9-14.4]; *P* < .001), and mean (SD) WIQ stair-climbing score (6.54 [22.7] vs −1.70 [30.3]; adjusted between-group difference: 10.6 [95% CI, 5.1-16.0]; *P* < .001) (Figure 2; eTable 3 in Supplement 1).

### Supervised Treadmill Exercise Compared With Home-Based Walking Exercise

Compared with supervised treadmill exercise, home-based walking exercise was associated with significantly improved mean (SD) 6MW distance (31.8 [6.4] m vs 55.6 [8.1] m; adjusted between-group difference: −23.8 m [95% CI, −44.0 to −3.6]; *P* = .021) (Table 2). Compared with supervised treadmill exercise, home-based walking exercise was associated with significantly less improvement in maximal treadmill walking distance (adjusted between-group difference: 132.5 m [95% CI, 72.1-192.9]; *P* < .001). Home-based walking exercise was associated with significantly improved mean (SD) WIQ speed score compared with supervised exercise (2.70 [2.40] vs 9.67 [2.41]; adjusted between-group difference: −6.97 m [95% CI, −13.6 to −0.3]) (Table 2). Home-based exercise improved the WIQ distance and stair-climbing scores more than supervised exercise, but differences did not reach statistical significance (Table 2).

**Table 2.** Treatment Outcome of Supervised Treadmill Exercise and Home-Based Walking Exercise Among Participants with PAD^a^

Outcome	No.	Adjusted between-group change, least squares mean (SE)		Adjusted between-group difference for supervised treadmill vs home-based exercise, least squares mean (95% CI)^b^	*P* value
		Supervised treadmill exercise vs nonexercise control	Home-based exercise vs nonexercise control		
6MW distance, m	635	31.78 (6.36)	55.59 (8.09)	−23.81 (−43.98 to −3.64)	.02
Maximum treadmill walking distance, m	570	186.16 (22.65)	53.65 (20.92)	132.51 (72.08 to 192.94)	<.001
Pain-free treadmill walking distance, m	570	99.04 (22.56)	42.02 (21.58)	57.02 (−4.17 to 118.21)	.07
WIQ distance score	616	5.27 (2.67)	10.50 (2.63)	−5.23 (−12.58 to 2.12)	.16
WIQ speed score	625	2.70 (2.40)	9.67 (2.41)	−6.97 (−13.64 to −0.30)	.04
WIQ stair-climbing score	623	4.00 (2.66)	10.56 (2.78)	−6.56 (−14.10 to 0.98)	.09

Abbreviations: 6MW, 6-minute walk; PAD, peripheral artery disease; WIQ, Walking Impairment Questionnaire (range, 0-100, with 100-indicating best).

^a^ A negative value favors home-based walking exercise. Individual participant data meta-analysis was performed using analysis of covariance adjusting for study, age, sex, race, smoking, myocardial infarction, heart failure, and baseline measure of outcome of interest to estimate the adjusted between-group difference in outcomes among those randomized to supervised treadmill exercise and to home-based walking exercise.

^b^ Results in this column were based on comparison of the estimates of treatment outcomes of supervised compared with home-based walking exercise from the previous 2 columns and does not represent a separate regression analysis.

## Discussion

In this individual participant data meta-analysis of 5 randomized clinical trials of exercise therapy for PAD, supervised treadmill exercise was associated with significantly improved 6MW distance by 31.8-m compared with control, and home-based walking exercise was associated with significantly improved 6MW distance by 55.6-m compared with control, adjusting for study, age, sex, race, smoking, history of myocardial infarction, heart failure, and baseline 6MW distance. In analyses limited to people with PAD randomized to either supervised or home-based walking exercise, home-based walking exercise was associated with significantly greater improvement in 6MW by 22.5 m compared with supervised treadmill exercise, while supervised treadmill exercise was associated with significantly greater improvement in maximal treadmill walking distance, by 132.2 m compared with home-based exercise.

Home-based walking exercise was associated with significant improvement in the WIQ distance, speed, and stair climbing scores compared with control. Supervised walking exercise was associated with significant improvement in the WIQ distance score, but not with significant improvement in the WIQ speed or stair climbing scores compared with control. In analyses limited to participants randomized to either supervised or home-based exercise, home-based exercise was associated with significantly greater improvement in the WIQ speed score compared with supervised exercise. Compared with supervised exercise, home-based walking exercise was associated with greater improvement in the WIQ distance and the WIQ stair-climbing scores, but these results did not reach statistical significance, perhaps due to inadequate statistical power. A prior study^18^ showed that compared with control walking exercise interventions were associated consistently with greater improvement in 6MW distance but were less consistently associated with patient-reported outcome measures of walking performance.

Gardner et al^6^ randomized 180 people with PAD to either supervised treadmill exercise, home-based walking exercise, or an attention control group for 12 weeks. At 12-week follow-up, supervised treadmill exercise and home-based walking exercise each significantly improved maximal treadmill walking time and claudication onset time compared with attention control. Supervised treadmill exercise improved maximal treadmill walking time significantly more than home-based walking exercise. However, home-based walking exercise improved 6MW distance significantly more than supervised treadmill exercise and significantly more than the control group, while supervised treadmill exercise had no significant effect on 6MW distance compared with control. Compared with the study by Gardner et al,^6^ the current study includes a larger sample size, multiple clinical trials, and longer follow-up. In contrast to results reported by Gardner et al,^6^ in the current analyses, supervised treadmill exercise significantly improved 6MW compared with the control group, with an effect size consistent with a clinically meaningful change. Vemulapalli et al^19^ conducted a meta-analysis that included 4 observational studies and 24 clinical trials that compared the associations of supervised and unsupervised exercise with change in treadmill walking distance in people with PAD. Supervised treadmill walking exercise was associated with significantly improved treadmill walking performance compared with unsupervised exercise, but there were no significant differences in quality-of-life measures between supervised and unsupervised exercise. Compared with the study by Vemulapalli et al,^19^ the current analyses included only randomized clinical trials and included the 6MW outcome. In a meta-analysis of 11 randomized clinical trials of 807 participants with PAD, Golledge et al^20^ reported that home-based exercise significantly improved treadmill walking performance, 6MW, and physical activity compared with control. The report by Golledge et al^20^ did not include clinical trials of supervised exercise or compare supervised and home-based exercise.

Supervised exercise is first line therapy for walking impairment in PAD and has been covered by Centers for Medicare & Medicaid Services for PAD since 2017.^1,2^ Few people with PAD participate in supervised walking exercise, due to inconvenience of regular travel to a facility for supervised exercise, lack of available exercise facilities, and the Centers for Medicare & Medicaid Services required co-pay.^4,21,22^ Home-based walking exercise circumvents these barriers. Data reported here demonstrated a large and consistent effect of home-based walking exercise on improved 6MW distance and also significantly improved the WIQ walking speed score compared with supervised treadmill exercise.

The home-based walking exercise interventions in these analyses were not intended to exactly replicate supervised exercise interventions but were designed to be as effective as possible. Participants randomized to home-based exercise were asked to walk for exercise 5 times per week, making walking exercise a near-daily behavior. While it is possible that 5 times weekly supervised exercise could attain larger improvements in 6MW distance than reported here, 5 times weekly exercise at an exercise center is not feasible for most people. Despite the efficacy of the home-based exercise intervention in the LITE Trial, participants randomized to high intensity home-based exercise walked for exercise just 2.9 days per week for a mean of 74.98 minutes per week.^11^

### Limitations

This study has several limitations. First, data were combined from different randomized clinical trials and comparisons reported here were not prespecified. Second, the randomized clinical trials of home-based exercise tested highly effective home-based exercise interventions with weekly monitoring and coaching feedback. Third, data were from clinical trials led by one investigative team. Fourth, the comparisons between supervised and home-based exercise lacked statistical power for the WIQ distance and stair-climbing measures.

## Conclusions

In 5 randomized clinical trials, home-based exercise was associated with significantly greater improvement in 6MW distance but significantly less improvement in maximal treadmill walking distance compared with supervised treadmill exercise. These findings support home-based walking exercise as a first-line therapy for walking limitations in PAD.

## Notes

Supplement 1. **eTable 1.** Supervised Treadmill Exercise and Home-Based Walking Exercise Clinical Trials Included in Analysis **eTable 2.** 6-Month Change in Study Outcomes Among Participants with PAD Randomized to Supervised Treadmill Exercise or Non-Exercise Control (n = 370) **eTable 3.** 6-Month Change in Study Outcomes Among Participants with PAD Randomized to Home-Based Walking Exercise or Non-Exercise Control (n = 349) **eTable 4.** Baseline Characteristics of Supervised Treadmill Exercise Trials by Study **eTable 5.** Baseline Characteristics of Home-Based Walking Exercise Trials by Study **eFigure 1.** Consort Diagram for Supervised Treadmill Exercise Randomized Clinical Trials Included in Post Hoc Analyses **eFigure 2.** Consort Diagram for Home-Based Walking Exercise Randomized Clinical Trials Included in Post Hoc Analyses

Supplement 2.
Data Sharing Statement
