# Supplementary material for: Home-Based Walking Exercise and Supervised Treadmill Exercise in Patients With Peripheral Artery Disease: An Individual Participant Data Meta-Analysis
Source: JAMA Netw Open. 2023 Sep 21;6(9):e2334590. doi: 10.1001/jamanetworkopen.2023.34590 (PMC10514734; doi:10.1001/jamanetworkopen.2023.34590)
Supplement: Supplement 1. — eTable 1. Supervised Treadmill Exercise and Home-Based Walking Exercise Clinical Trials Included in Analysis eTable 2. 6-Month Change in Study Outcomes Among Participants with PAD Randomized to Supervised Treadmill Exercise or Non-Exercise Control (n = 370) eTable 3. 6-Month Change in Study Outcomes Among Participants with PAD Randomized to Home-Based Walking Exercise or Non-Exercise Control (n = 349) eTable 4. Baseline Characteristics of Supervised Treadmill Exercise Trials by Study eTable 5. Baseline Characteristics of Home-Based Walking Exercise Trials by Study eFigure 1. Consort Diagram for Supervised Treadmill Exercise Randomized Clinical Trials Included in Post Hoc Analyses eFigure 2. Consort Diagram for Home-Based Walking Exercise Randomized Clinical Trials Included in Post Hoc Analyses [file jamanetwopen-e2334590-s001.pdf]

Supplemental Online Content

Thangada ND, Zhang D, Tian L, et al. Home-based walking exercise and supervised treadmill exercise in patients with peripheral artery disease: an individual participant data meta-analysis. *JAMA Netw Open.* 2023;6(9):e2334590. doi:10.1001/jamanetworkopen.2023.34590

eTable 1. Supervised Treadmill Exercise and Home-Based Walking Exercise Clinical Trials Included in Analysis

eTable 2. 6-Month Change in Study Outcomes Among Participants with PAD Randomized to Supervised Treadmill Exercise or Non-Exercise Control (n=370)

eTable 3. 6-Month Change in Study Outcomes Among Participants with PAD Randomized to Home-Based Walking Exercise or Non-Exercise Control (n=349)

eTable 4. Baseline Characteristics of Supervised Treadmill Exercise Trials by Study

eTable 5. Baseline Characteristics of Home-Based Walking Exercise Trials by Study

eFigure 1. Consort Diagram for Supervised Treadmill Exercise Randomized Clinical Trials Included in Post Hoc Analyses

eFigure 2. Consort Diagram for Home-Based Walking Exercise Randomized Clinical Trials Included in Post Hoc Analyses

This supplemental material has been provided by the authors to give readers additional information about their work.

© 2023 Thangada ND et al. *JAMA Network Open*.

Supplemental Table 1. Supervised Treadmill Exercise and Home-Based Walking Exercise Clinical Trials Included in Analysis

Trial Name	N	Sites/Dates	Primary Specific Aim	Randomization	Groups	Other
Study to Improve Leg Circulation (SILC)	156 participants with PAD	Chicago, IL<br><br>4/2004-8/2008	To determine whether supervised treadmill exercise and whether lower extremity strength training improved 6MW, compared to attention control.	Randomly permuted block method stratified by presence or absence of intermittent claudication.	1. Supervised treadmill exercise only<br>2. Supervised lower extremity resistance training only<br>3. Non-exercise control	Supervised exercise significantly improved six-minute walk distance and treadmill walking time.<br><br>Strength training did not significantly improve six-minute walk distance.
Effect of Granulocyte Stimulating Factor (GM-CSF) With or Without Supervised Exercise on Walking Performance in Patients with Peripheral Artery Disease (PROPEL)	210 participants with PAD	Chicago, IL<br><br>1/2012-12/2016	1.To determine whether GM-CSF combined with supervised treadmill exercise improved 6MW distance, compared with exercise alone and compared with GM-CSF alone.<br>2.To determine whether GM-CSF alone improved 6MW more than placebo and whether exercise improved 6MW more than an attention control.	Randomly permuted block method stratified by diabetes status. Block sizes were randomly selected from 8 and 12.	1.Supervised treadmill exercise plus GM-CSF<br>2. Supervised treadmill exercise plus placebo<br>3. Non-exercise control plus GM-CSF<br>4. Non-exercise control plus placebo	Supervised exercise significantly improved six-minute walk distance.<br><br>GM-CSF had no significant effect on 6MW.
Effect of Telmisartan on Walking Performance in Patients with Lower Extremity Peripheral Artery Disease (TELEX)	114 participants with PAD	Chicago, IL and New Orleans, LA<br><br>12/2015-11/2021	To determine whether telmisartan improves 6MWT compared to a placebo.	Randomly permuted block method stratified by consent to muscle biopsy. Block sizes randomly selected from 8 and 12.	1.Supervised treadmill exercise plus telmisartan<br>2. Supervised exercise plus placebo<br>3. Non-exercise control plus telmisartan<br>4. Non-exercise control plus placebo	Telmisartan had no significant effect on 6MW.
Group Oriented Arterial Leg Study to Improve Walking Performance in Patients with Peripheral Artery Disease (GOALS)	194 participants with PAD	Chicago, IL<br><br>11/2008-12/2012	To determine if a Group-Mediated Cognitive Behavioral intervention improves functional performance.	Randomly permuted block method stratified by baseline 6MW.	1.Home-based walking exercise<br>2. Non-exercise control	A Group-Mediated Cognitive Behavioral intervention significantly improved 6MW.
Effect of Low-Intensity vs High-Intensity Home-Based Walking Exercise on Walk Distance in Patients with Peripheral Artery Disease (LITE)	305 participants with PAD	Chicago, IL, New Orleans, LA, Pittsburgh, PA and Minneapolis, MN<br><br>9/2015-10/2020	To determine if a low-intensity walking exercise intervention, conducted at a pace without ischemic leg symptoms, improves 6MW compared to a high-intensity walking intervention, conducted at a pace with ischemic leg symptoms.	Randomly permuted block method with block sizes of 61 stratified by site and presence or absence of muscle biopsy.	1. Home-based walking exercise at low-intensity (pace not inducing ischemic leg symptoms)<br>2. Home-based walking exercise at high-intensity (pace inducing ischemic leg symptoms)<br>3. Non-exercise control	Only high-intensity home-based walking exercise was effective and significantly improved 6MW.

Supplemental Table 2. 6-Month Change in Study Outcomes Among Participants with PAD Randomized to Supervised Treadmill Exercise or Non-Exercise Control (n=370)

Outcome	Trial	N	6-Month Change in Outcome for Supervised Treadmill Exercise, mean (SE)	6-Month Change in Outcome for Non-Exercise Control, mean (SE)	Between Group Difference (95% CI)		P Value
					Unadjusted	Adjusted	
Six-minute walk distance (meters)	SILC	95	20.91 (7.44)	-15.02 (7.51)	35.93 (14.94, 56.92)	36.85 (15.15, 58.55)	0.0011
	PROPEL	161	34.26 (7.46)	-3.23 (7.41)	37.49 (16.73, 58.25)	39.35 (19.09, 59.62)	0.0002
	TELEX	80	18.15 (8.16)	-1.83 (8.16)	19.98 (-3.00, 42.96)	12.72 (-10.75, 36.18)	0.2836
	Meta analysis	336	---	---	32.88 (20.16, 45.60)	31.78 (19.32, 44.24)	<.0001
Maximum treadmill walking distance (meters)	SILC	92	218.05 (25.10)	35.46 (26.22)	182.59 (110.48, 254.70)	208.92 (134.32, 283.52)	<.0001
	PROPEL	156	260.15 (23.50)	39.96 (23.20)	220.20 (154.96, 285.44)	203.36 (137.47, 269.25)	<.0001
	TELEX	68	203.29 (37.77)	81.93 (35.61)	121.36 (17.72, 225.00)	117.11 (4.76, 229.47)	0.0413
	Meta analysis	316	---	---	188.03 (143.87, 232.19)	186.16 (141.77, 230.55)	<.0001
Pain-free treadmill walking distance (meters)	SILC	92	192.03 (26.30)	58.84 (27.47)	133.19 (57.64, 208.75)	133.94 (58.41, 209.47)	0.0007
	PROPEL	156	154.91 (20.30)	53.51 (20.04)	101.40 (45.04, 157.75)	107.68 (52.87, 162.49)	0.0002
	TELEX	68	176.96 (46.51)	118.42 (43.85)	58.54 (-69.09, 186.17)	40.61 (-89.87, 171.09)	0.5358
	Meta analysis	316	---	---	101.45 (57.11, 145.79)	99.04 (54.83, 143.25)	<.0001
WIQ distance score	SILC	75	17.97 (3.71)	4.19 (4.07)	13.79 (2.81, 24.77)	13.11 (2.04, 24.18)	0.0210
	PROPEL	161	7.61 (2.86)	5.71 (2.88)	1.90 (-6.12, 9.91)	3.48 (-4.40, 11.36)	0.3848
	TELEX	80	7.11 (3.50)	2.12 (3.50)	4.99 (-4.88, 14.85)	2.91 (-7.02, 12.85)	0.5606
	Meta analysis	316	---	---	5.48 (0.06, 10.90)	5.27 (0.03, 10.51)	0.0488
WIQ speed score	SILC	80	8.67 (3.45)	7.25 (3.81)	1.42 (-8.81, 11.66)	4.22 (-6.45, 14.90)	0.4325
	PROPEL	163	7.76 (2.70)	3.74 (2.69)	4.02 (-3.51, 11.55)	4.13 (-2.92, 11.17)	0.2493
	TELEX	80	3.02 (3.27)	2.09 (3.27)	0.92 (-8.29, 10.14)	-0.95 (-9.92, 8.02)	0.8341
	Meta analysis	323	---	---	2.61 (-2.44, 7.67)	2.70 (-2.01, 7.42)	0.2601
WIQ stair-climbing score	SILC	79	9.42 (3.71)	1.91 (3.96)	7.51 (-3.29, 18.31)	6.00 (-4.59, 16.58)	0.2622
	PROPEL	162	4.69 (3.01)	2.44 (2.97)	2.25 (-6.10, 10.60)	4.60 (-3.13, 12.32)	0.2415
	TELEX	80	7.29 (3.98)	1.77 (3.98)	5.52 (-5.69, 16.73)	2.98 (-7.74, 13.70)	0.5809
	Meta analysis	321	---	---	4.36 (-1.30, 10.01)	4.00 (-1.21, 9.21)	0.1319

Between group difference was estimated using ANCOVA adjusted for study, age, sex, race, smoking, myocardial infarction, heart failure, and baseline measure of outcome variable of interest for unadjusted and adjusted comparisons, respectively.

Supplemental Table 3. 6-Month Change in Study Outcomes Among Participants with PAD Randomized to Home-Based Walking Exercise or Non-Exercise Control (n=349)

Outcome	Trial	N	6-Month Change in Outcome for Home-Based Walking Exercise, mean (SE)	6-Month Change in Outcome for Non-Exercise Control, mean (SE)	Between Group Difference (95% CI)		P value
					Unadjusted	Adjusted	
Six-minute walk distance (meters)	GOALS	169	42.73 (7.63)	-11.69 (7.59)	54.42 (33.17, 75.66)	59.98 (39.29, 80.68)	<.0001
	LITE	130	34.55 (7.30)	-10.91 (10.04)	45.45 (20.89, 70.02)	51.41 (25.96, 76.85)	0.0001
	Meta analysis	299	---	---	50.74 (34.76, 66.72)	55.59 (39.73, 71.46)	<.0001
Maximum treadmill walking distance (meters)	GOALS	164	89.26 (18.51)	33.03 (18.51)	56.23 (4.54, 107.92)	52.36 (-0.27, 104.99)	0.0512
	LITE	90	97.78 (19.36)	38.60 (26.70)	59.18 (-6.36, 124.72)	52.69 (-15.80, 121.17)	0.1297
	Meta analysis	254	---	---	57.21 (16.74, 97.67)	53.65 (12.65, 94.64)	0.0105
Pain-free treadmill walking distance (meters)	GOALS	164	89.26 (18.51)	33.03 (18.51)	49.98 (6.33, 93.63)	42.75 (-0.86, 86.36)	0.0546
	LITE	90	97.78 (19.36)	38.60 (26.70)	29.44 (-67.59, 126.46)	54.92 (-37.39, 147.23)	0.2400
	Meta analysis	254	---	---	43.17 (-0.34, 86.69)	42.02 (-0.28, 84.32)	0.0515
WIQ distance score	GOALS	167	11.61 (2.59)	1.45 (2.61)	10.16 (2.91, 17.42)	11.16 (4.35, 17.97)	0.0015
	LITE	133	8.72 (2.45)	1.15 (1.15)	7.57 (-0.58, 15.72)	9.84 (1.61, 18.06)	0.0195
	Meta analysis	300	---	---	9.07 (3.68, 14.46)	10.50 (5.35, 15.65)	<.0001
WIQ speed score	GOALS	169	11.57 (2.61)	1.84 (2.59)	9.73 (2.47, 16.98)	8.68 (2.21, 15.15)	0.0088
	LITE	133	7.13 (2.32)	-4.95 (3.14)	12.08 (4.36, 19.80)	10.52 (3.40, 17.63)	0.0041
	Meta analysis	302	---	---	10.71 (5.44, 15.99)	9.67 (4.94, 14.40)	<.0001
WIQ stair-climbing score	GOALS	169	8.73 (2.96)	0.10 (2.94)	8.63 (0.39, 16.87)	9.30 (2.36, 16.25)	0.0090
	LITE	133	4.41 (2.71)	-4.96 (3.67)	9.37 (0.35, 18.40)	12.67 (3.78, 21.56)	0.0056
	Meta analysis	302	---	---	8.94 (2.89, 15.00)	10.56 (5.11, 16.01)	0.0002

Between group difference was estimated using ANCOVA adjusted for study, age, sex, race, smoking, myocardial infarction, heart failure, and baseline measure of outcome variable of interest for unadjusted and adjusted comparisons, respectively.

Supplemental Table 4. Baseline Characteristics of Supervised Treadmill Exercise Trials by Study

	SILC			PROPEL			TELEX		
**Baseline variable**	**Supervised exercise (N=51)**	**Control (N=53)**	**P value**	**Supervised exercise (N=87)**	**Control (N=89)**	**P value**	**Supervised exercise (N=44)**	**Control (N=46)**	**P value**
Age (years), mean (SD)	71.67 (8.68)	68.51 (11.87)	0.1258	66.62 (8.89)	66.10 (7.29)	0.6718	67.20 (9.87)	66.50 (10.93)	0.7494
Male, n (%)	24 (47.06)	25 (47.17)	0.9910	53 (60.92)	56 (62.92)	0.7845	25 (56.82)	28 (60.87)	0.6962
African American, n (%)	21 (41.18)	26 (49.06)	0.4195	55 (63.22)	66 (74.16)	0.1175	28 (63.64)	32 (69.57)	0.5509
Ankle brachial index, mean (SD)	0.61 (0.18)	0.60 (0.18)	0.7520	0.69 (0.18)	0.70 (0.18)	0.6682	0.75 (0.23)	0.74 (0.26)	0.8902
Body mass index (kg/m^2), mean (SD)	29.93 (6.16)	30.42 (7.12)	0.7070	30.97 (6.25)	30.71 (7.03)	0.7974	29.27 (7.53)	28.56 (6.14)	0.6279
Current smoker, n (%)	11 (21.57)	17 (32.08)	0.2272	35 (40.23)	26 (29.21)	0.1247	22 (50.00)	25 (54.35)	0.6798
Myocardial infarction, n (%)	12 (24.00)	7 (13.21)	0.1581	16 (18.39)	22 (24.72)	0.3077	5 (11.36)	10 (21.74)	0.1867
Heart failure, n (%)	4 (8.16)	9 (16.98)	0.1821	8 (9.20)	14 (15.73)	0.1900	1 (2.27)	5 (10.87)	0.1022
Stroke, n (%)	11 (22.00)	10 (18.87)	0.6933	13 (14.94)	18 (20.22)	0.3577	8 (18.18)	4 (8.70)	0.1857
Angina, n (%)	6 (11.76)	4 (7.69)	0.4852	15 (17.24)	18 (20.22)	0.6122	5 (11.36)	2 (4.35)	0.2141
Pulmonary disease, n (%)	3 (5.88)	9 (17.65)	0.0652	11 (12.64)	10 (11.24)	0.7733	10 (22.73)	11 (23.91)	0.8942
Cancer, n (%)	12 (23.53)	8 (15.09)	0.2752	16 (18.39)	15 (16.85)	0.7890	9 (20.45)	7 (15.22)	0.5160
Diabetes, n (%)	20 (39.22)	25 (47.17)	0.4131	33 (37.93)	33 (37.08)	0.9070	16 (36.36)	13 (28.26)	0.4109
IC (Intermittent claudication), n (%)	18 (35.29)	20 (37.74)	0.6702	31 (35.63)	22 (24.72)	0.2357	4 (9.09)	6 (13.04)	0.5837
Leg pain not IC, n (%)	29 (56.86)	31 (58.49)		52 (59.77)	64 (71.91)		36 (81.82)	38 (82.61)	
Asymptomatic leg pain, n (%)	4 (7.84)	2 (3.77)		4 (4.60)	3 (3.37)		4 (9.09)	2 (4.35)	
Six-minute walk distance (meters), mean (SD)	327.80 (86.96)	316.58 (83.20)	0.5026	330.81 (102.47)	336.24 (98.38)	0.7203	316.51 (118.99)	359.92 (110.00)	0.0756
Total treadmill distance (meters), mean (SD)	383.02 (241.07)	322.54 (204.14)	0.1698	339.92 (267.62)	383.96 (235.89)	0.2482	330.17 (202.99)	438.19 (294.64)	0.0468
Treadmill distance at onset of leg symptom (meters), mean (SD)	202.46 (212.87)	155.78 (116.23)	0.1661	184.40 (204.59)	183.92 (185.49)	0.9871	149.10 (127.42)	238.54 (241.27)	0.0316
WIQ distance score, mean (SD)	26.04 (19.09)	30.52 (24.01)	0.3236	31.41 (25.99)	32.12 (25.12)	0.8554	37.74 (34.82)	38.28 (27.08)	0.9339

© 2023 Thangada ND et al. *JAMA Network Open*.

WIQ speed score, mean (SD)	32.10 (22.88)	27.87 (18.01)	0.3127	34.36 (24.72)	35.16 (24.51)	0.8288	34.76 (25.67)	39.11 (24.54)	0.4134
WIQ stair-climbing score, mean (SD)	41.03 (24.66)	42.24 (24.86)	0.8112	49.27 (29.92)	45.88 (28.31)	0.4419	44.98 (30.55)	51.99 (29.93)	0.2744

Supplemental Table 5. Baseline Characteristics of Home-Based Walking Exercise Trials by Study

	GOALS			LITE		
**Baseline variable**	**Home-based exercise (N=92)**	**Control (N=92)**	**P value**	**Home-based exercise (N=106)**	**Control (N=59)**	**P value**
Age (years), mean (SD)	69.09 (9.45)	71.15 (9.69)	0.1449	68.80 (8.50)	69.75 (9.91)	0.5206
Male, n (%)	47 (51.09)	45 (48.91)	0.7681	52 (49.06)	30 (50.85)	0.8255
African American, n (%)	51 (55.43)	41 (44.57)	0.1404	65 (61.32)	30 (50.85)	0.1920
Ankle brachial index, mean (SD)	0.67 (0.16)	0.68 (0.18)	0.7822	0.73 (0.23)	0.78 (0.29)	0.2253
Body mass index (kg/m^2), mean (SD)	29.00 (7.03)	28.85 (6.58)	0.8812	31.34 (7.55)	30.37 (6.81)	0.4142
Current smoker, n (%)	24 (26.09)	20 (21.74)	0.4894	21 (19.81)	12 (20.34)	0.9353
Myocardial infarction, n (%)	13 (14.13)	13 (14.13)	1.0000	28 (26.42)	6 (10.17)	0.0134
Heart failure, n (%)	9 (9.78)	10 (10.87)	0.8086	19 (17.92)	7 (11.86)	0.3058
Stroke, n (%)	9 (9.78)	13 (14.13)	0.3634	25 (23.58)	19 (32.20)	0.2302
Angina, n (%)	14 (15.22)	15 (16.48)	0.8146	20 (18.87)	10 (16.95)	0.7594
Pulmonary disease, n (%)	13 (14.13)	13 (14.13)	1.0000	17 (16.04)	9 (15.25)	0.8947
Cancer, n (%)	15 (16.30)	14 (15.38)	0.8647	19 (17.92)	16 (27.12)	0.1662
Diabetes, n (%)	26 (28.26)	34 (36.96)	0.2084	46 (43.40)	30 (50.85)	0.3574
IC (Intermittent claudication), n (%)	30 (32.61)	20 (21.74)	0.2140	19 (17.92)	11 (18.64)	0.8416
Leg pain not IC, n (%)	54 (58.70)	65 (70.65)		82 (77.36)	44 (74.58)	
Asymptomatic leg pain, n (%)	8 (8.70)	7 (7.61)		5 (4.72)	4 (6.78)	
Six-minute walk distance (meters), mean (SD)	355.16 (96.52)	352.41 (92.40)	0.8434	325.07 (101.56)	329.79 (85.86)	0.7635
Total treadmill distance (meters), mean (SD)	404.22 (249.14)	378.05 (256.58)	0.4835	352.16 (243.57)	403.06 (232.54)	0.1929
Treadmill distance at onset of leg symptom (meters), mean (SD)	163.63 (141.18)	188.28 (202.05)	0.3387	156.11 (157.20)	154.13 (129.73)	0.9346
WIQ distance score, mean (SD)	34.25 (27.50)	32.15 (26.23)	0.5979	32.42 (25.93)	36.03 (26.18)	0.3942
WIQ speed score, mean (SD)	34.91 (23.50)	33.72 (23.14)	0.7290	34.68 (22.87)	40.05 (24.59)	0.1619
WIQ stair-climbing score, mean (SD)	47.83 (25.79)	46.29 (25.55)	0.6846	47.52 (27.37)	45.20 (24.50)	0.5880

Supplemental Figure 1. Consort Diagram for Supervised Treadmill Exercise Randomized Clinical Trials Included in Post Hoc Analyses

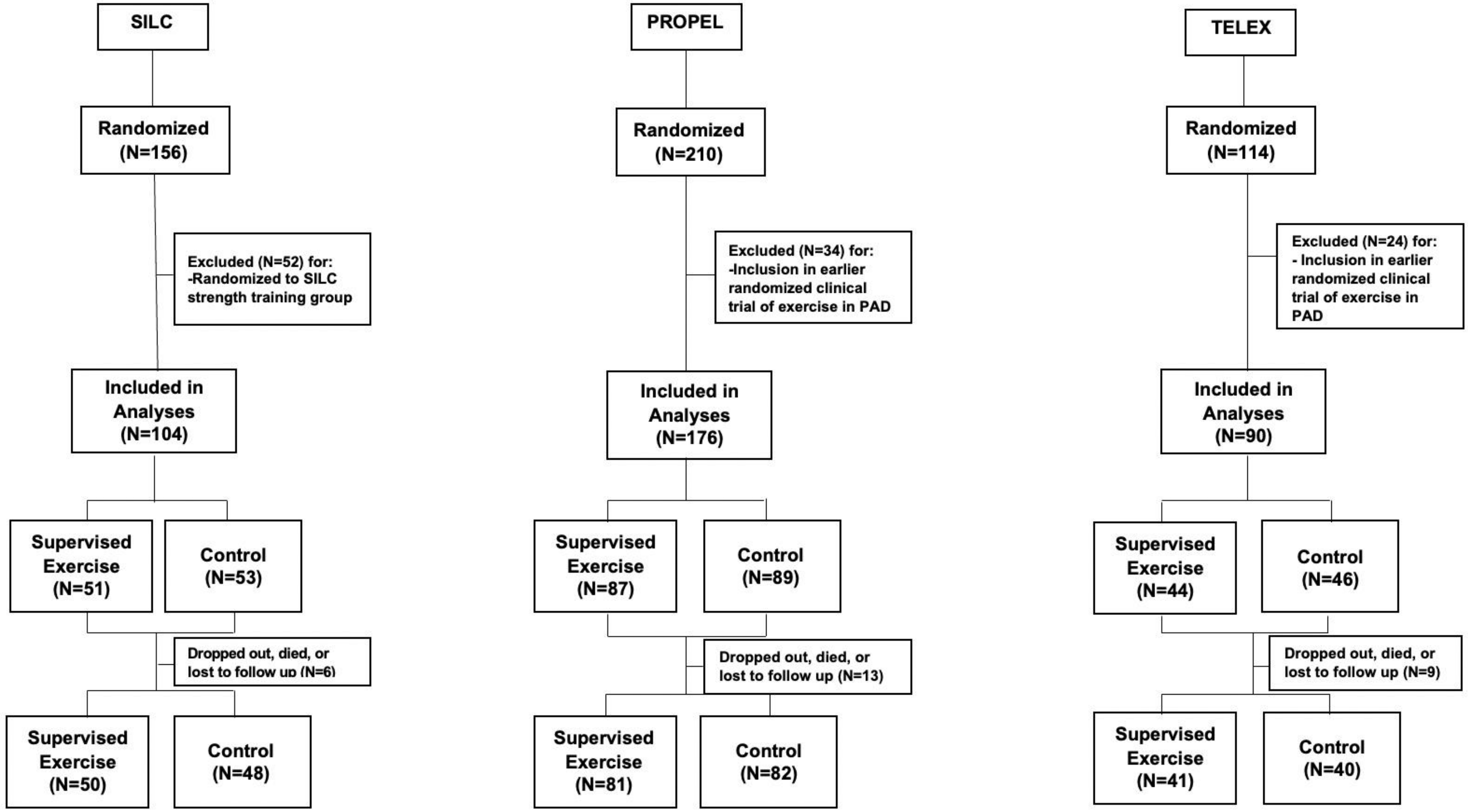

Supplemental Figure 2. Consort Diagram for Home-Based Walking Exercise Randomized Clinical Trials Included in Post Hoc Analyses

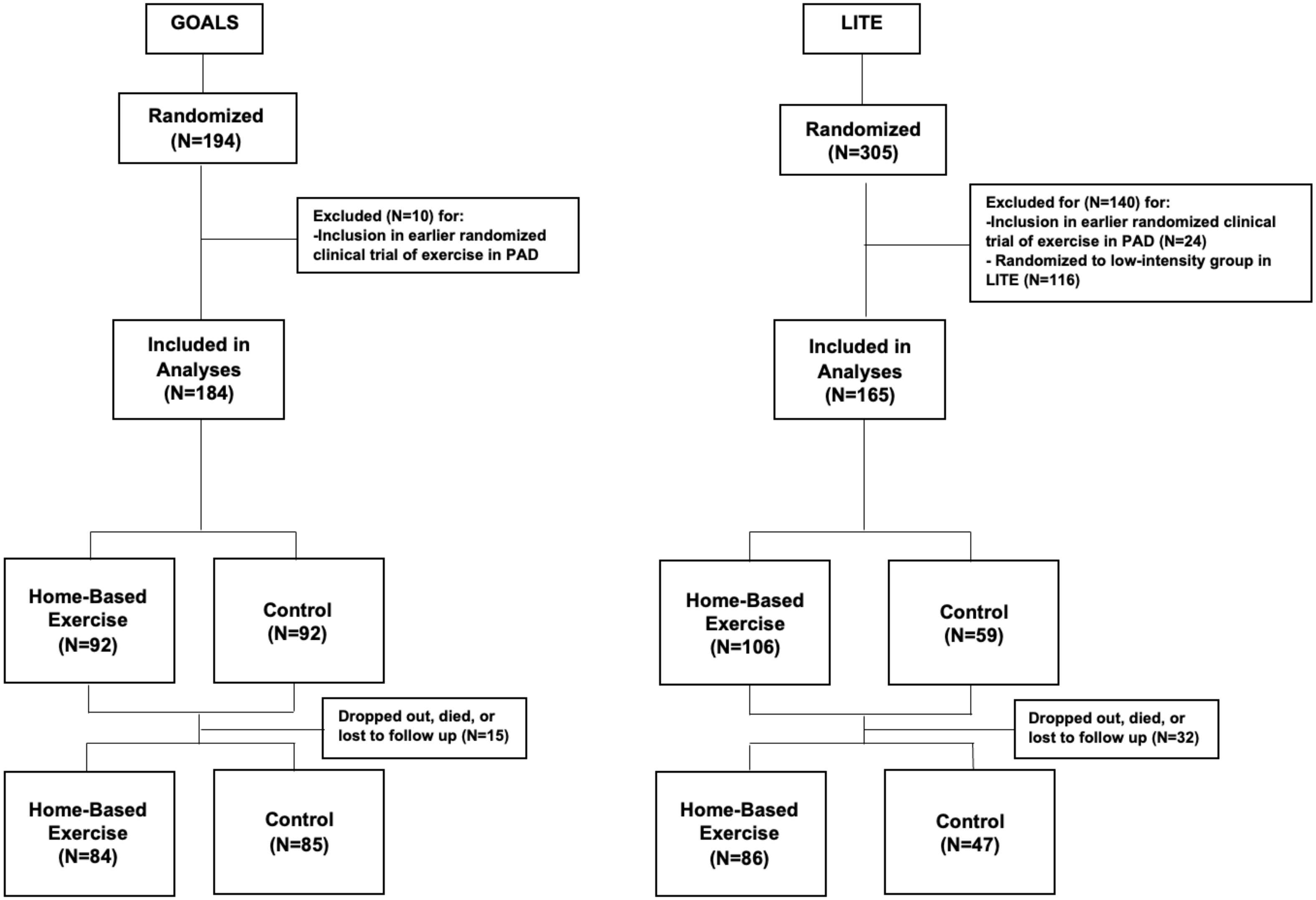

© 2023 Thangada ND et al. *JAMA Network Open*.

SAS code and results for JNO revision

1. Table 1

SAS code:

```
**Read in analyses dataset;
data master;
set 'location\dataset';
run;

**supervised exercise groups vs home based exercise groups;
data exercise;
set master;
if group1 ^= 'Control';
if study in ('1 SILC' '3 PROPEL' '5 TELEX') then study2 = 'supervised';
if study in ('2 GOALS' '4 LITE') then study2 = 'home-based';
run;

proc glm data = exercise;
class study2;
model age abi0 bmi0 dist6min_m0 t_dist_meter0 on_dist_meter0 distscr0
speedscr0 climbscr0 = study2/solution;
means study2;
run;

proc NPAR1WAY data=exercise wilcoxon;
class study2;
var age abi0 bmi0 dist6min_m0 t_dist_meter0 on_dist_meter0 distscr0 speedscr0
climbscr0;
run;

proc freq data = exercise;
table (gender aarace cur_smoker0 MI0 CHF0 stroke0 angina0 pulmdx0 cancer0
diabetes0 leg_sym_bv)*study2/norow nopercent chisq;
run;

**supervised studies control vs home based studies control;
data control;
set master;
if group1 = 'Control';
if study in ('1 SILC' '3 PROPEL' '5 TELEX') then study2 = 'supervised';
if study in ('2 GOALS' '4 LITE') then study2 = 'home-based';
run;
proc glm data = control;
class study2;
model age abi0 bmi0 dist6min_m0 t_dist_meter0 on_dist_meter0 distscr0
speedscr0 climbscr0 = study2/solution;
means study2;
run;

proc NPAR1WAY data=control wilcoxon;
class study2;
var age abi0 bmi0 dist6min_m0 t_dist_meter0 on_dist_meter0 distscr0 speedscr0
climbscr0;
run;
```

```
proc freq data = control;
table (gender aarace cur_smoker0 MI0 CHF0 stroke0 angina0 pulmdx0 cancer0
diabetes0 leg_sym_bv)*study2/norow nopercent chisq;
run;
```

2. Table 2

Data in Current Table 2 can be found in Supplemental Table 2 and 3, 7th column of each table.

3. Supplemental Table 2

Illustrative SAS code:

```
%macro ana2(indata=, var=, name=, blv=);
title 'Group mean and stddev';
proc means data = &indata n mean stddev maxdec=2;
class group2;
var &var;
run;

title 'Unadjusted';
proc glm data=&indata;
class group2(ref='Control') %if &indata=sup_con %or &indata=home_con %then
%do; study %end;;
model &var = group2 %if &indata=sup_con %or &indata=home_con %then %do; study
%end;/solution;
lsmeans group2/tdiff pdiff cl  STDERR;
ods output "Type III Model ANOVA"=a1 LSMeanCL=a2 LSMeanDiffCL=a3;
run;


title 'Adjusted IPD';
proc glm data=&indata;
class group2(ref='Control') gender aarace(ref='0') cur_smoker0(ref='0')
MI0(ref='0') CHF0(ref='0') %if &indata=sup_con %or &indata=home_con %then
%do; study %end;;
model &var = group2 &blv age gender aarace cur_smoker0 MI0 CHF0 %if
&indata=sup_con %or &indata=home_con %then %do; study %end;/solution;
lsmeans group2/tdiff pdiff cl stderr ;
ods output "Type III Model ANOVA"=b1 LSMeanCL=b2 LSMeanDiffCL=b3;
run;

%mend;

**example: six-minute walk outcome;
%ana2(indata=SILC, var=ch_dist6min_m60, name=dist6min, blv=dist6min_m0);
%ana2(indata=PROPEL, var=ch_dist6min_m60, name=dist6min, blv=dist6min_m0);
%ana2(indata=TELEX, var=ch_dist6min_m60, name=dist6min, blv=dist6min_m0);
%ana2(indata=sup_con, var=ch_dist6min_m60, name=dist6min, blv=dist6min_m0);
```

SAS output results:

```
**six-minute walk;

**SILC;
```

Group mean and stddev

The MEANS Procedure

Analysis Variable : ch_dist6min_m60				
**group2**	**N Obs**	**N**	**Mean**	**Std Dev**
**Control**	**53**	47	-15.02	55.54
**Supervised exercise**	**51**	48	20.91	47.25

Unadjusted

The GLM Procedure
Least Squares Means

group2	ch_dist6min_m60 LSMEAN	Standard Error	H0:LSMEAN=0	H0:LSMean1=LSMean2	
			Pr > |t|	t Value	Pr > |t|
**Supervised exercise**	20.9105500	7.4356001	0.0060	3.40	0.0010
**Control**	-15.0195064	7.5142859	0.0486		

group2	ch_dist6min_m60 LSMEAN	95% Confidence Limits	
**Supervised exercise**	20.910550	6.144922	35.676178
**Control**	-15.019506	-29.941389	-0.097624

Least Squares Means for Effect group2				
**i**	**j**	**Difference Between Means**	**95% Confidence Limits for LSMean(i)-LSMean(j)**	
**1**	**2**	35.930056	14.937525	56.922587

Adjusted IPD

The GLM Procedure
Least Squares Means

group2	ch_dist6min_m60 LSMEAN	Standard Error	H0:LSMEAN=0	H0:LSMean1=LSMean2	
			Pr > |t|	t Value	Pr > |t|
**Supervised exercise**	17.7721179	11.8785475	0.1384	3.38	0.0011

group2	ch_dist6min_m60 LSMEAN	Standard Error	H0:LSMEAN=0	H0:LSMean1=LSMean2	
			Pr > |t|	t Value	Pr > |t|
**Control**	-19.0773777	10.7064336	0.0784		

group2	ch_dist6min_m60 LSMEAN	95% Confidence Limits	
**Supervised exercise**	17.772118	-5.853835	41.398071
**Control**	-19.077378	-40.372043	2.217288

Least Squares Means for Effect group2				
i	j	Difference Between Means	95% Confidence Limits for LSMean(i)-LSMean(j)	
**1**	**2**	36.849496	15.153298	58.545693

```
**PROPEL;
```

Group mean and stddev

The MEANS Procedure

Analysis Variable : ch_dist6min_m60				
group2	N Obs	N	Mean	Std Dev
**Control**	**89**	81	-3.23	65.89
**Supervised exercise**	**87**	80	34.26	67.50

Unadjusted

The GLM Procedure
Least Squares Means

group2	ch_dist6min_m60 LSMEAN	Standard Error	H0:LSMEAN=0	H0:LSMean1=LSMean2	
			Pr > |t|	t Value	Pr > |t|
**Supervised exercise**	34.2633300	7.4563248	<.0001	3.57	0.0005
**Control**	-3.2286222	7.4101552	0.6636		

group2	ch_dist6min_m60 LSMEAN	95% Confidence Limits	
**Supervised exercise**	34.263330	19.537117	48.989543
**Control**	-3.228622	-17.863651	11.406406

Least Squares Means for Effect group2				
**i**	**j**	**Difference Between Means**	**95% Confidence Limits for LSMean(i)-LSMean(j)**	
**1**	**2**	37.491952	16.730319	58.253585

Adjusted IPD

The GLM Procedure
Least Squares Means

group2	ch_dist6min_m60 LSMEAN	Standard Error	H0:LSMEAN=0	H0:LSMean1=LSMean2	
			Pr > |t|	t Value	Pr > |t|
**Supervised exercise**	27.1791493	10.2575741	0.0089	3.84	0.0002
**Control**	-12.1756805	10.4980489	0.2479		

group2	ch_dist6min_m60 LSMEAN	95% Confidence Limits	
**Supervised exercise**	27.179149	6.913322	47.444976
**Control**	-12.175681	-32.916612	8.565251

Least Squares Means for Effect group2				
**i**	**j**	**Difference Between Means**	**95% Confidence Limits for LSMean(i)-LSMean(j)**	
**1**	**2**	39.354830	19.093540	59.616119

```
**TELEX;
```

Group mean and stddev

The MEANS Procedure

Analysis Variable : ch_dist6min_m60				
**group2**	**N Obs**	**N**	**Mean**	**Std Dev**
**Control**	**46**	40	-1.83	47.96
**Supervised exercise**	**44**	40	18.15	55.04

Unadjusted

The GLM Procedure
Least Squares Means

group2	ch_dist6min_m60 LSMEAN	Standard Error	H0:LSMEAN=0	H0:LSMean1=LSMean2	
			Pr > |t|	t Value	Pr > |t|
**Supervised exercise**	18.1508400	8.1622239	0.0291	1.73	0.0874
**Control**	-1.8288000	8.1622239	0.8233		

group2	ch_dist6min_m60 LSMEAN	95% Confidence Limits	
**Supervised exercise**	18.150840	1.901101	34.400579
**Control**	-1.828800	-18.078539	14.420939

Least Squares Means for Effect group2				
i	j	Difference Between Means	95% Confidence Limits for LSMean(i)-LSMean(j)	
**1**	**2**	19.979640	-3.000962	42.960242

Adjusted IPD

The GLM Procedure
Least Squares Means

group2	ch_dist6min_m60 LSMEAN	Standard Error	H0:LSMEAN=0	H0:LSMean1=LSMean2	
			Pr > |t|	t Value	Pr > |t|
**Supervised exercise**	12.2948901	13.8369938	0.3772	1.08	0.2836
**Control**	-0.4212325	12.5255288	0.9733		

group2	ch_dist6min_m60 LSMEAN	95% Confidence Limits	
**Supervised exercise**	12.294890	-15.295292	39.885072
**Control**	-0.421232	-25.396427	24.553963

Least Squares Means for Effect group2				
i	j	Difference Between Means	95% Confidence Limits for LSMean(i)-LSMean(j)	
**1**	**2**	12.716123	-10.749754	36.181999

```
**Supervised exercise studies combined;
```

Unadjusted

The GLM Procedure
Least Squares Means

group2	ch_dist6min_m60 LSMEAN	Standard Error	H0:LSMEAN=0	H0:LSMean1=LSMean2	
			Pr > |t|	t Value	Pr > |t|
**Supervised exercise**	25.3154911	4.6730288	<.0001	5.08	<.0001
**Control**	-7.5650747	4.6794608	0.1069		

group2	ch_dist6min_m60 LSMEAN	95% Confidence Limits	
**Supervised exercise**	25.315491	16.123012	34.507970
**Control**	-7.565075	-16.770206	1.640057

Least Squares Means for Effect group2				
i	j	Difference Between Means	95% Confidence Limits for LSMean(i)-LSMean(j)	
**1**	**2**	32.880566	20.159176	45.601956

Adjusted IPD

The GLM Procedure
Least Squares Means

group2	ch_dist6min_m60 LSMEAN	Standard Error	H0:LSMEAN=0	H0:LSMean1=LSMean2	
			Pr > |t|	t Value	Pr > |t|
**Supervised exercise**	22.4175819	6.8342505	0.0012	5.02	<.0001
**Control**	-9.3621383	6.3868042	0.1437		

group2	ch_dist6min_m60 LSMEAN	95% Confidence Limits	
**Supervised exercise**	22.417582	8.972161	35.863003
**Control**	-9.362138	-21.927272	3.202996

Least Squares Means for Effect group2				
i	j	Difference Between Means	95% Confidence Limits for LSMean(i)-LSMean(j)	
**1**	**2**	31.779720	19.323990	44.235451

© 2023 Thangada ND et al. *JAMA Network Open*.

```
**maximal treadmill walking distance;

**SILC;
```

Group mean and stddev

The MEANS Procedure

Analysis Variable : ch_t_dist_meter60				
**group2**	**N Obs**	**N**	**Mean**	**Std Dev**
**Control**	**53**	44	35.46	138.00
**Supervised exercise**	**51**	48	218.05	201.23

Unadjusted

The GLM Procedure
Least Squares Means

group2	ch_t_dist_meter60 LSMEAN	Standard Error	H0:LSMEAN=0	H0:LSMean1=LSMean2	
			Pr > |t|	t Value	Pr > |t|
**Supervised exercise**	218.052531	25.102027	<.0001	5.03	<.0001
**Control**	35.463392	26.218212	0.1796		

group2	ch_t_dist_meter60 LSMEAN	95% Confidence Limits	
**Supervised exercise**	218.052531	168.182974	267.922089
**Control**	35.463392	-16.623662	87.550446

Least Squares Means for Effect group2				
**i**	**j**	**Difference Between Means**	**95% Confidence Limits for LSMean(i)-LSMean(j)**	
**1**	**2**	182.589140	110.477879	254.700400

Adjusted IPD

The GLM Procedure
Least Squares Means

group2	ch_t_dist_meter60 LSMEAN	Standard Error	H0:LSMEAN=0	H0:LSMean1=LSMean2	
			Pr > |t|	t Value	Pr > |t|
**Supervised exercise**	221.595803	40.459354	<.0001	5.57	<.0001
**Control**	12.679290	38.707628	0.7441		

group2	ch_t_dist_meter60 LSMEAN	95% Confidence Limits	
**Supervised exercise**	221.595803	141.079123	302.112483
**Control**	12.679290	-64.351344	89.709924

Least Squares Means for Effect group2				
i	j	Difference Between Means	95% Confidence Limits for LSMean(i)-LSMean(j)	
**1**	**2**	208.916513	134.315857	283.517169

```
**PROPEL;
```

Group mean and stddev

The MEANS Procedure

Analysis Variable : ch_t_dist_meter60				
group2	N Obs	N	Mean	Std Dev
**Control**	**89**	79	39.96	171.21
**Supervised exercise**	**87**	77	260.15	236.83

Unadjusted

The GLM Procedure
Least Squares Means

group2	ch_t_dist_meter60 LSMEAN	Standard Error	H0:LSMEAN=0	H0:LSMean1=LSMean2	
			Pr > |t|	t Value	Pr > |t|
**Supervised exercise**	260.154057	23.500780	<.0001	6.67	<.0001
**Control**	39.956322	23.201394	0.0870		

group2	ch_t_dist_meter60 LSMEAN	95% Confidence Limits	
**Supervised exercise**	260.154057	213.728548	306.579567
**Control**	39.956322	-5.877756	85.790400

© 2023 Thangada ND et al. *JAMA Network Open*.

Least Squares Means for Effect group2				
**i**	**j**	**Difference Between Means**	**95% Confidence Limits for LSMean(i)-LSMean(j)**	
**1**	**2**	220.197735	154.959015	285.436455

Adjusted IPD

The GLM Procedure
Least Squares Means

**group2**	**ch_t_dist_meter60 LSMEAN**	**Standard Error**	**H0:LSMEAN=0**	**H0:LSMean1=LSMean2**	
			**Pr > |t|**	**t Value**	**Pr > |t|**
**Supervised exercise**	217.645000	33.233450	<.0001	6.10	<.0001
**Control**	14.284411	33.672480	0.6720		

**group2**	**ch_t_dist_meter60 LSMEAN**	**95% Confidence Limits**	
**Supervised exercise**	217.645000	151.967949	283.322051
**Control**	14.284411	-52.260266	80.829088

Least Squares Means for Effect group2				
**i**	**j**	**Difference Between Means**	**95% Confidence Limits for LSMean(i)-LSMean(j)**	
**1**	**2**	203.360588	137.467755	269.253422

```
**TELEX;
```

Group mean and stddev

The MEANS Procedure

Analysis Variable : ch_t_dist_meter60				
**group2**	**N Obs**	**N**	**Mean**	**Std Dev**
**Control**	**46**	36	81.93	220.66
**Supervised exercise**	**44**	32	203.29	205.45

Unadjusted

The GLM Procedure

Least Squares Means

group2	ch_t_dist_meter60 LSMEAN	Standard Error	H0:LSMEAN=0	H0:LSMean1=LSMean2	
			Pr > |t|	t Value	Pr > |t|
**Supervised exercise**	203.291440	37.768603	<.0001	2.34	0.0224
**Control**	81.932498	35.608580	0.0246		

group2	ch_t_dist_meter60 LSMEAN	95% Confidence Limits	
**Supervised exercise**	203.291440	127.883991	278.698889
**Control**	81.932498	10.837673	153.027322

Least Squares Means for Effect group2				
i	j	Difference Between Means	95% Confidence Limits for LSMean(i)-LSMean(j)	
**1**	**2**	121.358942	17.721317	224.996568

Adjusted IPD

The GLM Procedure
Least Squares Means

group2	ch_t_dist_meter60 LSMEAN	Standard Error	H0:LSMEAN=0	H0:LSMean1=LSMean2	
			Pr > |t|	t Value	Pr > |t|
**Supervised exercise**	146.035452	67.254660	0.0339	2.09	0.0413
**Control**	28.920878	61.459789	0.6397		

group2	ch_t_dist_meter60 LSMEAN	95% Confidence Limits	
**Supervised exercise**	146.035452	11.459189	280.611715
**Control**	28.920878	-94.059875	151.901631

Least Squares Means for Effect group2				
i	j	Difference Between Means	95% Confidence Limits for LSMean(i)-LSMean(j)	
**1**	**2**	117.114574	4.762297	229.466850

Least Squares Means for Effect group2				
i	j	Difference Between Means	95% Confidence Limits for LSMean(i)-LSMean(j)	
**1**	**2**	121.358942	17.721317	224.996568

```
**Supervised exercise studies combined;
```

Unadjusted

The GLM Procedure
Least Squares Means

group2	ch_t_dist_meter60 LSMEAN	Standard Error	H0:LSMEAN=0	H0:LSMean1=LSMean2	
			Pr > |t|	t Value	Pr > |t|
**Supervised exercise**	234.368505	16.418598	<.0001	8.38	<.0001
**Control**	46.339189	16.273923	0.0047		

group2	ch_t_dist_meter60 LSMEAN	95% Confidence Limits	
**Supervised exercise**	234.368505	202.063329	266.673682
**Control**	46.339189	14.318675	78.359703

Least Squares Means for Effect group2				
i	j	Difference Between Means	95% Confidence Limits for LSMean(i)-LSMean(j)	
**1**	**2**	188.029317	143.866442	232.192191

Adjusted IPD

The GLM Procedure
Least Squares Means

group2	ch_t_dist_meter60 LSMEAN	Standard Error	H0:LSMEAN=0	H0:LSMean1=LSMean2	
			Pr > |t|	t Value	Pr > |t|
**Supervised exercise**	212.161062	23.978051	<.0001	8.25	<.0001
**Control**	26.000442	22.837839	0.2558		

group2	ch_t_dist_meter60 LSMEAN	95% Confidence Limits	
**Supervised exercise**	212.161062	164.975849	259.346275
**Control**	26.000442	-18.941005	70.941889

Least Squares Means for Effect group2				
i	j	Difference Between Means	95% Confidence Limits for LSMean(i)-LSMean(j)	
**1**	**2**	186.160620	141.771092	230.550148

© 2023 Thangada ND et al. *JAMA Network Open*.

```
**pain-free treadmill walking distance;

**SILC;
```

Group mean and stddev

The MEANS Procedure

Analysis Variable : ch_on_dist_meter60				
**group2**	**N Obs**	**N**	**Mean**	**Std Dev**
**Control**	**53**	44	58.84	146.45
**Supervised exercise**	**51**	48	192.03	209.67

Unadjusted

The GLM Procedure
Least Squares Means

group2	ch_on_dist_meter60 LSMEAN	Standard Error	H0:LSMEAN=0	H0:LSMean1=LSMean2	
			Pr > |t|	t Value	Pr > |t|
**Supervised exercise**	192.031143	26.301548	<.0001	3.50	0.0007
**Control**	58.836414	27.471070	0.0349		

group2	ch_on_dist_meter60 LSMEAN	95% Confidence Limits	
**Supervised exercise**	192.031143	139.778528	244.283758
**Control**	58.836414	4.260337	113.412490

Least Squares Means for Effect group2				
**i**	**j**	**Difference Between Means**	**95% Confidence Limits for LSMean(i)-LSMean(j)**	
**1**	**2**	133.194729	57.637573	208.751885

Adjusted IPD

The GLM Procedure
Least Squares Means

group2	ch_on_dist_meter60 LSMEAN	Standard Error	H0:LSMEAN=0	H0:LSMean1=LSMean2	
			Pr > |t|	t Value	Pr > |t|
**Supervised exercise**	102.798545	41.202466	0.0147	3.53	0.0007

group2	ch_on_dist_meter60 LSMEAN	Standard Error	H0:LSMEAN=0	H0:LSMean1=LSMean2	
			Pr > |t|	t Value	Pr > |t|
**Control**	-31.139252	38.850983	0.4252		

group2	ch_on_dist_meter60 LSMEAN	95% Confidence Limits	
**Supervised exercise**	102.798545	20.803024	184.794066
**Control**	-31.139252	-108.455172	46.176668

Least Squares Means for Effect group2				
i	j	Difference Between Means	95% Confidence Limits for LSMean(i)-LSMean(j)	
**1**	**2**	133.937796	58.405758	209.469835

```
**PROPEL;
```

Group mean and stddev

The MEANS Procedure

Analysis Variable : ch_on_dist_meter60				
group2	N Obs	N	Mean	Std Dev
**Control**	**89**	79	53.51	160.68
**Supervised exercise**	**87**	77	154.91	194.45

Unadjusted

The GLM Procedure
Least Squares Means

group2	ch_on_dist_meter60 LSMEAN	Standard Error	H0:LSMEAN=0	H0:LSMean1=LSMean2	
			Pr > |t|	t Value	Pr > |t|
**Supervised exercise**	154.908069	20.301686	<.0001	3.55	0.0005
**Control**	53.511820	20.043055	0.0084		

group2	ch_on_dist_meter60 LSMEAN	95% Confidence Limits	
**Supervised exercise**	154.908069	114.802331	195.013807
**Control**	53.511820	13.917004	93.106636

Least Squares Means for Effect group2				
**i**	**j**	**Difference Between Means**	**95% Confidence Limits for LSMean(i)-LSMean(j)**	
**1**	**2**	101.396249	45.038289	157.754209

Adjusted IPD

The GLM Procedure
Least Squares Means

group2	ch_on_dist_meter60 LSMEAN	Standard Error	H0:LSMEAN=0	H0:LSMean1=LSMean2	
			**Pr > |t|**	**t Value**	**Pr > |t|**
**Supervised exercise**	142.635733	27.549988	<.0001	3.88	0.0002
**Control**	34.952864	28.208949	0.2173		

group2	ch_on_dist_meter60 LSMEAN	95% Confidence Limits	
**Supervised exercise**	142.635733	88.190530	197.080937
**Control**	34.952864	-20.794600	90.700328

Least Squares Means for Effect group2				
**i**	**j**	**Difference Between Means**	**95% Confidence Limits for LSMean(i)-LSMean(j)**	
**1**	**2**	107.682869	52.871827	162.493912

```
**TELEX;
```

Group mean and stddev

The MEANS Procedure

Analysis Variable : ch_on_dist_meter60				
**group2**	**N Obs**	**N**	**Mean**	**Std Dev**
**Control**	**46**	36	118.42	282.93
**Supervised exercise**	**44**	32	176.96	238.75

Unadjusted

The GLM Procedure
Least Squares Means

group2	ch_on_dist_meter60 LSMEAN	Standard Error	H0:LSMEAN=0	H0:LSMean1=LSMean2	
			Pr > |t|	t Value	Pr > |t|
**Supervised exercise**	176.957990	46.511273	0.0003	0.92	0.3631
**Control**	118.415929	43.851249	0.0088		

group2	ch_on_dist_meter60 LSMEAN	95% Confidence Limits	
**Supervised exercise**	176.957990	84.095237	269.820743
**Control**	118.415929	30.864086	205.967772

Least Squares Means for Effect group2				
i	j	Difference Between Means	95% Confidence Limits for LSMean(i)-LSMean(j)	
**1**	**2**	58.542061	-69.085585	186.169707

Adjusted IPD

The GLM Procedure
Least Squares Means

group2	ch_on_dist_meter60 LSMEAN	Standard Error	H0:LSMEAN=0	H0:LSMean1=LSMean2	
			Pr > |t|	t Value	Pr > |t|
**Supervised exercise**	116.422152	77.313143	0.1374	0.62	0.5358
**Control**	75.812735	71.104830	0.2907		

group2	ch_on_dist_meter60 LSMEAN	95% Confidence Limits	
**Supervised exercise**	116.422152	-38.281089	271.125393
**Control**	75.812735	-66.467702	218.093172

Least Squares Means for Effect group2				
i	j	Difference Between Means	95% Confidence Limits for LSMean(i)-LSMean(j)	
**1**	**2**	40.609417	-89.866942	171.085777

```
**Supervised exercise studies combined;
```

Unadjusted

The GLM Procedure
Least Squares Means

group2	ch_on_dist_meter60 LSMEAN	Standard Error	H0:LSMEAN=0	H0:LSMean1=LSMean2	
			Pr > |t|	t Value	Pr > |t|
**Supervised exercise**	177.151056	16.483949	<.0001	4.50	<.0001
**Control**	75.704331	16.338698	<.0001		

group2	ch_on_dist_meter60 LSMEAN	95% Confidence Limits	
**Supervised exercise**	177.151056	144.717296	209.584817
**Control**	75.704331	43.556367	107.852296

Least Squares Means for Effect group2				
i	j	Difference Between Means	95% Confidence Limits for LSMean(i)-LSMean(j)	
**1**	**2**	101.446725	57.108070	145.785380

Adjusted IPD

The GLM Procedure
Least Squares Means

group2	ch_on_dist_meter60 LSMEAN	Standard Error	H0:LSMEAN=0	H0:LSMean1=LSMean2	
			Pr > |t|	t Value	Pr > |t|
**Supervised exercise**	147.835788	23.923366	<.0001	4.41	<.0001
**Control**	48.794695	22.766929	0.0329		

group2	ch_on_dist_meter60 LSMEAN	95% Confidence Limits	
**Supervised exercise**	147.835788	100.758186	194.913390
**Control**	48.794695	3.992788	93.596601

Least Squares Means for Effect group2				
i	j	Difference Between Means	95% Confidence Limits for LSMean(i)-LSMean(j)	
**1**	**2**	99.041093	54.829091	143.253096

```
**WIQ distance score;

**SILC;
```

Group mean and stddev

The MEANS Procedure

Analysis Variable : ch_distscr60				
**group2**	**N Obs**	**N**	**Mean**	**Std Dev**
**Control**	**53**	34	4.19	18.25
**Supervised exercise**	**51**	41	17.97	27.47

Unadjusted

The GLM Procedure
Least Squares Means

group2	ch_distscr60 LSMEAN	Standard Error	H0:LSMEAN=0	H0:LSMean1=LSMean2	
			Pr > |t|	t Value	Pr > |t|
**Supervised exercise**	17.9739468	3.7091512	<.0001	2.50	0.0146
**Control**	4.1861631	4.0731182	0.3075		

group2	ch_distscr60 LSMEAN	95% Confidence Limits	
**Supervised exercise**	17.973947	10.581619	25.366275
**Control**	4.186163	-3.931550	12.303876

Least Squares Means for Effect group2				
**i**	**j**	**Difference Between Means**	**95% Confidence Limits for LSMean(i)-LSMean(j)**	
**1**	**2**	13.787784	2.808541	24.767026

Adjusted IPD

The GLM Procedure
Least Squares Means

group2	ch_distscr60 LSMEAN	Standard Error	H0:LSMEAN=0	H0:LSMean1=LSMean2	
			Pr > |t|	t Value	Pr > |t|
**Supervised exercise**	14.9796420	5.9962449	0.0151	2.37	0.0210
**Control**	1.8698434	6.1843878	0.7634		

group2	ch_distscr60 LSMEAN	95% Confidence Limits	
**Supervised exercise**	14.979642	3.000766	26.958518
**Control**	1.869843	-10.484892	14.224578

© 2023 Thangada ND et al. *JAMA Network Open*.

Least Squares Means for Effect group2				
**i**	**j**	**Difference Between Means**	**95% Confidence Limits for LSMean(i)-LSMean(j)**	
**1**	**2**	13.109799	2.042448	24.177149

```
**PROPEL;
```

Group mean and stddev

The MEANS Procedure

Analysis Variable : ch_distscr60				
**group2**	**N Obs**	**N**	**Mean**	**Std Dev**
**Control**	**89**	80	5.71	26.46
**Supervised exercise**	**87**	81	7.61	25.01

Unadjusted

The GLM Procedure
Least Squares Means

**group2**	**ch_distscr60 LSMEAN**	**Standard Error**	**H0:LSMEAN=0**	**H0:LSMean1=LSMean2**	
			**Pr > |t|**	**t Value**	**Pr > |t|**
**Supervised exercise**	7.60820006	2.85955215	0.0086	0.47	0.6410
**Control**	5.71289062	2.87736884	0.0488		

**group2**	**ch_distscr60 LSMEAN**	**95% Confidence Limits**	
**Supervised exercise**	7.608200	1.960595	13.255805
**Control**	5.712891	0.030098	11.395683

Least Squares Means for Effect group2				
**i**	**j**	**Difference Between Means**	**95% Confidence Limits for LSMean(i)-LSMean(j)**	
**1**	**2**	1.895309	-6.116530	9.907149

Adjusted IPD

The GLM Procedure
Least Squares Means

group2	ch_distscr60 LSMEAN	Standard Error	H0:LSMEAN=0	H0:LSMean1=LSMean2	
			Pr > |t|	t Value	Pr > |t|
**Supervised exercise**	6.96951036	4.05784531	0.0879	0.87	0.3848
**Control**	3.49299552	4.18780650	0.4055		

group2	ch_distscr60 LSMEAN	95% Confidence Limits	
**Supervised exercise**	6.969510	-1.047550	14.986571
**Control**	3.492996	-4.780828	11.766819

Least Squares Means for Effect group2				
i	j	Difference Between Means	95% Confidence Limits for LSMean(i)-LSMean(j)	
**1**	**2**	3.476515	-4.404469	11.357499

```
**TELEX;
```

Group mean and stddev

The MEANS Procedure

Analysis Variable : ch_distscr60				
group2	N Obs	N	Mean	Std Dev
**Control**	**46**	40	2.12	22.14
**Supervised exercise**	**44**	40	7.11	22.17

Unadjusted

The GLM Procedure
Least Squares Means

group2	ch_distscr60 LSMEAN	Standard Error	H0:LSMEAN=0	H0:LSMean1=LSMean2	
			Pr > |t|	t Value	Pr > |t|
**Supervised exercise**	7.10759943	3.50301841	0.0459	1.01	0.3172
**Control**	2.12002841	3.50301841	0.5468		

group2	ch_distscr60 LSMEAN	95% Confidence Limits	
**Supervised exercise**	7.107599	0.133625	14.081573
**Control**	2.120028	-4.853946	9.094002

Least Squares Means for Effect group2				
i	j	Difference Between Means	95% Confidence Limits for LSMean(i)-LSMean(j)	
1	2	4.987571	-4.875118	14.850260

Adjusted IPD

The GLM Procedure
Least Squares Means

group2	ch_distscr60 LSMEAN	Standard Error	H0:LSMEAN=0	H0:LSMean1=LSMean2	
			Pr > |t|	t Value	Pr > |t|
Supervised exercise	2.36965789	5.97144340	0.6927	0.58	0.5606
Control	-0.54457782	5.34757121	0.9192		

group2	ch_distscr60 LSMEAN	95% Confidence Limits	
Supervised exercise	2.369658	-9.537062	14.276378
Control	-0.544578	-11.207332	10.118176

Least Squares Means for Effect group2				
i	j	Difference Between Means	95% Confidence Limits for LSMean(i)-LSMean(j)	
1	2	2.914236	-7.023106	12.851578

```
**Supervised exercise studies combined;
```

Unadjusted

The GLM Procedure
Least Squares Means

group2	ch_distscr60 LSMEAN	Standard Error	H0:LSMEAN=0	H0:LSMean1=LSMean2	
			Pr > |t|	t Value	Pr > |t|
Supervised exercise	10.3187538	1.9753143	<.0001	1.99	0.0474
Control	4.8351108	2.0380306	0.0183		

group2	ch_distscr60 LSMEAN	95% Confidence Limits	
Supervised exercise	10.318754	6.432132	14.205375
Control	4.835111	0.825089	8.845133

Least Squares Means for Effect group2				
**i**	**j**	**Difference Between Means**	**95% Confidence Limits for LSMean(i)-LSMean(j)**	
**1**	**2**	5.483643	0.064490	10.902797

Adjusted IPD

The GLM Procedure
Least Squares Means

group2	ch_distscr60 LSMEAN	Standard Error	H0:LSMEAN=0	H0:LSMean1=LSMean2	
			**Pr > |t|**	**t Value**	**Pr > |t|**
**Supervised exercise**	9.15566127	2.87717319	0.0016	1.98	0.0488
**Control**	3.88670781	2.80360442	0.1667		

group2	ch_distscr60 LSMEAN	95% Confidence Limits	
**Supervised exercise**	9.155661	3.493891	14.817432
**Control**	3.886708	-1.630293	9.403708

Least Squares Means for Effect group2				
**i**	**j**	**Difference Between Means**	**95% Confidence Limits for LSMean(i)-LSMean(j)**	
**1**	**2**	5.268953	0.028003	10.509904

```
**WIQ speed score;

**SILC;
```

Group mean and stddev

The MEANS Procedure

Analysis Variable : ch_speedscr60				
**group2**	**N Obs**	**N**	**Mean**	**Std Dev**
**Control**	**53**	36	7.25	22.10
**Supervised exercise**	**51**	44	8.67	23.48

Unadjusted

© 2023 Thangada ND et al. *JAMA Network Open*.

The GLM Procedure
Least Squares Means

group2	ch_speedscr60 LSMEAN	Standard Error	H0:LSMEAN=0	H0:LSMean1=LSMean2	
			Pr > |t|	t Value	Pr > |t|
**Supervised exercise**	8.67094862	3.44730790	0.0139	0.28	0.7824
**Control**	7.24637681	3.81114228	0.0609		

group2	ch_speedscr60 LSMEAN	95% Confidence Limits	
**Supervised exercise**	8.670949	1.807886	15.534011
**Control**	7.246377	-0.341025	14.833778

Least Squares Means for Effect group2				
i	j	Difference Between Means	95% Confidence Limits for LSMean(i)-LSMean(j)	
**1**	**2**	1.424572	-8.806278	11.655422

Adjusted IPD

The GLM Procedure
Least Squares Means

group2	ch_speedscr60 LSMEAN	Standard Error	H0:LSMEAN=0	H0:LSMean1=LSMean2	
			Pr > |t|	t Value	Pr > |t|
**Supervised exercise**	9.13487543	5.49632743	0.1011	0.79	0.4325
**Control**	4.91099898	5.54802127	0.3792		

group2	ch_speedscr60 LSMEAN	95% Confidence Limits	
**Supervised exercise**	9.134875	-1.832875	20.102626
**Control**	4.910999	-6.159905	15.981903

Least Squares Means for Effect group2				
i	j	Difference Between Means	95% Confidence Limits for LSMean(i)-LSMean(j)	
**1**	**2**	4.223876	-6.451664	14.899416

```
**PROPEL;
```

Group mean and stddev

The MEANS Procedure

Analysis Variable : ch_speedscr60				
**group2**	**N Obs**	**N**	**Mean**	**Std Dev**
**Control**	**89**	82	3.74	25.46
**Supervised exercise**	**87**	81	7.76	23.13

Unadjusted

The GLM Procedure
Least Squares Means

group2	ch_speedscr60 LSMEAN	Standard Error	H0:LSMEAN=0	H0:LSMean1=LSMean2	
			Pr > |t|	t Value	Pr > |t|
**Supervised exercise**	7.75630703	2.70344372	0.0047	1.05	0.2934
**Control**	3.73806999	2.68690874	0.1661		

group2	ch_speedscr60 LSMEAN	95% Confidence Limits	
**Supervised exercise**	7.756307	2.417524	13.095090
**Control**	3.738070	-1.568059	9.044199

Least Squares Means for Effect group2				
**i**	**j**	**Difference Between Means**	**95% Confidence Limits for LSMean(i)-LSMean(j)**	
**1**	**2**	4.018237	-3.508888	11.545362

Adjusted IPD

The GLM Procedure
Least Squares Means

group2	ch_speedscr60 LSMEAN	Standard Error	H0:LSMEAN=0	H0:LSMean1=LSMean2	
			Pr > |t|	t Value	Pr > |t|
**Supervised exercise**	5.94523474	3.62873399	0.1034	1.16	0.2493
**Control**	1.81904478	3.70573496	0.6242		

group2	ch_speedscr60 LSMEAN	95% Confidence Limits	
**Supervised exercise**	5.945235	-1.223286	13.113755
**Control**	1.819045	-5.501590	9.139680

© 2023 Thangada ND et al. *JAMA Network Open*.

Least Squares Means for Effect group2				
i	j	Difference Between Means	95% Confidence Limits for LSMean(i)-LSMean(j)	
**1**	**2**	4.126190	-2.921802	11.174182

```
**TELEX;
```

Group mean and stddev

The MEANS Procedure

Analysis Variable : ch_speedscr60				
group2	N Obs	N	Mean	Std Dev
**Control**	**46**	40	2.09	19.11
**Supervised exercise**	**44**	40	3.02	22.16

Unadjusted

The GLM Procedure
Least Squares Means

group2	ch_speedscr60 LSMEAN	Standard Error	H0:LSMEAN=0	H0:LSMean1=LSMean2	
			Pr > |t|	t Value	Pr > |t|
**Supervised exercise**	3.01630435	3.27163716	0.3594	0.20	0.8422
**Control**	2.09239130	3.27163716	0.5243		

group2	ch_speedscr60 LSMEAN	95% Confidence Limits	
**Supervised exercise**	3.016304	-3.497025	9.529634
**Control**	2.092391	-4.420938	8.605721

Least Squares Means for Effect group2				
i	j	Difference Between Means	95% Confidence Limits for LSMean(i)-LSMean(j)	
**1**	**2**	0.923913	-8.287326	10.135152

Adjusted IPD

The GLM Procedure
Least Squares Means

group2	ch_speedscr60 LSMEAN	Standard Error	H0:LSMEAN=0	H0:LSMean1=LSMean2	
			Pr > |t|	t Value	Pr > |t|
**Supervised exercise**	2.69315360	5.32595935	0.6147	-0.21	0.8341
**Control**	3.63903181	4.75121433	0.4463		

group2	ch_speedscr60 LSMEAN	95% Confidence Limits	
**Supervised exercise**	2.693154	-7.926508	13.312815
**Control**	3.639032	-5.834620	13.112684

Least Squares Means for Effect group2				
i	j	Difference Between Means	95% Confidence Limits for LSMean(i)-LSMean(j)	
**1**	**2**	-0.945878	-9.916750	8.024994

```
**Supervised exercise studies combined;
```

Unadjusted

The GLM Procedure
Least Squares Means

group2	ch_speedscr60 LSMEAN	Standard Error	H0:LSMEAN=0	H0:LSMean1=LSMean2	
			Pr > |t|	t Value	Pr > |t|
**Supervised exercise**	6.70501351	1.84089490	0.0003	1.02	0.3101
**Control**	4.09265118	1.89617610	0.0316		

group2	ch_speedscr60 LSMEAN	95% Confidence Limits	
**Supervised exercise**	6.705014	3.083185	10.326842
**Control**	4.092651	0.362060	7.823242

Least Squares Means for Effect group2				
i	j	Difference Between Means	95% Confidence Limits for LSMean(i)-LSMean(j)	
**1**	**2**	2.612362	-2.443114	7.667839

Adjusted IPD

The GLM Procedure
Least Squares Means

group2	ch_speedscr60 LSMEAN	Standard Error	H0:LSMEAN=0	H0:LSMean1=LSMean2	
			Pr > |t|	t Value	Pr > |t|
**Supervised exercise**	6.12747885	2.55467413	0.0171	1.13	0.2601
**Control**	3.42346562	2.45745176	0.1646		

group2	ch_speedscr60 LSMEAN	95% Confidence Limits	
**Supervised exercise**	6.127479	1.100721	11.154237
**Control**	3.423466	-1.411991	8.258922

Least Squares Means for Effect group2				
i	j	Difference Between Means	95% Confidence Limits for LSMean(i)-LSMean(j)	
**1**	**2**	2.704013	-2.012067	7.420094

```
**WIQ stair-climbing score;

**SILC;
```

Group mean and stddev

The MEANS Procedure

Analysis Variable : ch_climbscr60				
group2	N Obs	N	Mean	Std Dev
**Control**	**53**	37	1.91	19.97
**Supervised exercise**	**51**	42	9.42	27.14

Unadjusted

The GLM Procedure
Least Squares Means

group2	ch_climbscr60 LSMEAN	Standard Error	H0:LSMEAN=0	H0:LSMean1=LSMean2	
			Pr > |t|	t Value	Pr > |t|
**Supervised exercise**	9.42460317	3.71242559	0.0131	1.38	0.1702
**Control**	1.91441441	3.95531923	0.6298		

group2	ch_climbscr60 LSMEAN	95% Confidence Limits	
**Supervised exercise**	9.424603	2.032219	16.816987
**Control**	1.914414	-5.961632	9.790461

Least Squares Means for Effect group2				
**i**	**j**	**Difference Between Means**	**95% Confidence Limits for LSMean(i)-LSMean(j)**	
**1**	**2**	7.510189	-3.291638	18.312015

Adjusted IPD

The GLM Procedure
Least Squares Means

group2	ch_climbscr60 LSMEAN	Standard Error	H0:LSMEAN=0	H0:LSMean1=LSMean2	
			Pr > |t|	t Value	Pr > |t|
**Supervised exercise**	9.87736488	5.59454537	0.0820	1.13	0.2622
**Control**	3.88022755	5.49335900	0.4824		

group2	ch_climbscr60 LSMEAN	95% Confidence Limits	
**Supervised exercise**	9.877365	-1.289394	21.044124
**Control**	3.880228	-7.084563	14.845018

Least Squares Means for Effect group2				
**i**	**j**	**Difference Between Means**	**95% Confidence Limits for LSMean(i)-LSMean(j)**	
**1**	**2**	5.997137	-4.589669	16.583944

```
**PROPEL;
```

Group mean and stddev

The MEANS Procedure

Analysis Variable : ch_climbscr60				
**group2**	**N Obs**	**N**	**Mean**	**Std Dev**
**Control**	**89**	82	2.44	28.05
**Supervised exercise**	**87**	80	4.69	25.67

Unadjusted

The GLM Procedure
Least Squares Means

group2	ch_climbscr60 LSMEAN	Standard Error	H0:LSMEAN=0	H0:LSMean1=LSMean2	
			Pr > |t|	t Value	Pr > |t|
**Supervised exercise**	4.68750000	3.00748046	0.1211	0.53	0.5955
**Control**	2.43902439	2.97057747	0.4128		

group2	ch_climbscr60 LSMEAN	95% Confidence Limits	
**Supervised exercise**	4.687500	-1.251978	10.626978
**Control**	2.439024	-3.427574	8.305622

Least Squares Means for Effect group2				
i	j	Difference Between Means	95% Confidence Limits for LSMean(i)-LSMean(j)	
**1**	**2**	2.248476	-6.099840	10.596791

Adjusted IPD

The GLM Procedure
Least Squares Means

group2	ch_climbscr60 LSMEAN	Standard Error	H0:LSMEAN=0	H0:LSMean1=LSMean2	
			Pr > |t|	t Value	Pr > |t|
**Supervised exercise**	-0.39507833	3.94044777	0.9203	1.18	0.2415
**Control**	-4.99351795	4.07223287	0.2220		

group2	ch_climbscr60 LSMEAN	95% Confidence Limits	
**Supervised exercise**	-0.395078	-8.179789	7.389632
**Control**	-4.993518	-13.038582	3.051546

Least Squares Means for Effect group2				
i	j	Difference Between Means	95% Confidence Limits for LSMean(i)-LSMean(j)	
**1**	**2**	4.598440	-3.128050	12.324929

```
**TELEX;
```

Group mean and stddev

The MEANS Procedure

© 2023 Thangada ND et al. *JAMA Network Open*.

Analysis Variable : ch_climbscr60				
**group2**	**N Obs**	**N**	**Mean**	**Std Dev**
**Control**	**46**	40	1.77	24.87
**Supervised exercise**	**44**	40	7.29	25.47

Unadjusted

The GLM Procedure
Least Squares Means

group2	ch_climbscr60 LSMEAN	Standard Error	H0:LSMEAN=0	H0:LSMean1=LSMean2	
			Pr > |t|	t Value	Pr > |t|
**Supervised exercise**	7.29166667	3.98039497	0.0708	0.98	0.3297
**Control**	1.77083333	3.98039497	0.6576		

group2	ch_climbscr60 LSMEAN	95% Confidence Limits	
**Supervised exercise**	7.291667	-0.632691	15.216024
**Control**	1.770833	-6.153524	9.695191

Least Squares Means for Effect group2				
**i**	**j**	**Difference Between Means**	**95% Confidence Limits for LSMean(i)-LSMean(j)**	
**1**	**2**	5.520833	-5.685901	16.727567

Adjusted IPD

The GLM Procedure
Least Squares Means

group2	ch_climbscr60 LSMEAN	Standard Error	H0:LSMEAN=0	H0:LSMean1=LSMean2	
			Pr > |t|	t Value	Pr > |t|
**Supervised exercise**	1.71319020	6.39335894	0.7895	0.55	0.5809
**Control**	-1.26782209	5.77372069	0.8268		

group2	ch_climbscr60 LSMEAN	95% Confidence Limits	
**Supervised exercise**	1.713190	-11.034805	14.461186
**Control**	-1.267822	-12.780294	10.244650

Least Squares Means for Effect group2				
**i**	**j**	**Difference Between Means**	**95% Confidence Limits for LSMean(i)-LSMean(j)**	
**1**	**2**	2.981012	-7.737089	13.699113

```
**Supervised exercise studies combined;
```

Unadjusted

The GLM Procedure
Least Squares Means

group2	ch_climbscr60 LSMEAN	Standard Error	H0:LSMEAN=0	H0:LSMean1=LSMean2	
			Pr > |t|	t Value	Pr > |t|
**Supervised exercise**	6.80361934	2.07515121	0.0012	1.51	0.1309
**Control**	2.44761153	2.10872164	0.2466		

group2	ch_climbscr60 LSMEAN	95% Confidence Limits	
**Supervised exercise**	6.803619	2.720810	10.886429
**Control**	2.447612	-1.701247	6.596470

Least Squares Means for Effect group2				
**i**	**j**	**Difference Between Means**	**95% Confidence Limits for LSMean(i)-LSMean(j)**	
**1**	**2**	4.356008	-1.302279	10.014294

Adjusted IPD

The GLM Procedure
Least Squares Means

group2	ch_climbscr60 LSMEAN	Standard Error	H0:LSMEAN=0	H0:LSMean1=LSMean2	
			Pr > |t|	t Value	Pr > |t|
**Supervised exercise**	3.31476034	2.81827314	0.2404	1.51	0.1319
**Control**	-0.68595012	2.70334512	0.7999		

group2	ch_climbscr60 LSMEAN	95% Confidence Limits	
**Supervised exercise**	3.314760	-2.230816	8.860336
**Control**	-0.685950	-6.005380	4.633480

Least Squares Means for Effect group2				
i	j	Difference Between Means	95% Confidence Limits for LSMean(i)-LSMean(j)	
1	2	4.000710	-1.210601	9.212022

4. Supplemental Table 3

Illustrative SAS code:

```
%macro ana2(indata=, var=, name=, blv=);
title 'Group mean and stddev';
proc means data = &indata n mean stddev maxdec=2;
class group2;
var &var;
run;

title 'Unadjusted';
proc glm data=&indata;
class group2(ref='Control') %if &indata=sup_con %or &indata=home_con %then
%do; study %end;;
model &var = group2 %if &indata=sup_con %or &indata=home_con %then %do; study
%end;/solution;
lsmeans group2/tdiff pdiff cl  STDERR;
ods output "Type III Model ANOVA"=a1 LSMeanCL=a2 LSMeanDiffCL=a3;
run;


title 'Adjusted IPD';
proc glm data=&indata;
class group2(ref='Control') gender aarace(ref='0') cur_smoker0(ref='0')
MI0(ref='0') CHF0(ref='0') %if &indata=sup_con %or &indata=home_con %then
%do; study %end;;
model &var = group2 &blv age gender aarace cur_smoker0 MI0 CHF0 %if
&indata=sup_con %or &indata=home_con %then %do; study %end;/solution;
lsmeans group2/tdiff pdiff cl stderr ;
ods output "Type III Model ANOVA"=b1 LSMeanCL=b2 LSMeanDiffCL=b3;
run;

%mend;

**example: six-minute walk outcome;
%ana2(indata=GOALS, var=ch_dist6min_m60, name=dist6min, blv=dist6min_m0);
%ana2(indata=LITE, var=ch_dist6min_m60, name=dist6min, blv=dist6min_m0);
%ana2(indata=home_con, var=ch_dist6min_m60, name=dist6min, blv=dist6min_m0);
```

SAS output results:

```
**six-minute walk;

**GOALS;
```

Group mean and stddev

The MEANS Procedure

Analysis Variable : ch_dist6min_m60				
**group2**	**N Obs**	**N**	**Mean**	**Std Dev**
**Control**	**92**	85	-11.69	70.62
**Home-based exercise**	**92**	84	42.73	69.26

Unadjusted

The GLM Procedure
Least Squares Means

group2	ch_dist6min_m60 LSMEAN	Standard Error	H0:LSMEAN=0	H0:LSMean1=LSMean2	
			Pr > |t|	t Value	Pr > |t|
**Home-based exercise**	42.7264286	7.6316710	<.0001	5.06	<.0001
**Control**	-11.6899765	7.5866460	0.1252		

group2	ch_dist6min_m60 LSMEAN	95% Confidence Limits	
**Home-based exercise**	42.726429	27.659442	57.793415
**Control**	-11.689976	-26.668071	3.288118

Least Squares Means for Effect group2				
**i**	**j**	**Difference Between Means**	**95% Confidence Limits for LSMean(i)-LSMean(j)**	
**1**	**2**	54.416405	33.171231	75.661579

Adjusted IPD

The GLM Procedure
Least Squares Means

group2	ch_dist6min_m60 LSMEAN	Standard Error	H0:LSMEAN=0	H0:LSMean1=LSMean2	
			Pr > |t|	t Value	Pr > |t|
**Home-based exercise**	36.7303210	11.5057889	0.0017	5.73	<.0001
**Control**	-23.2545825	11.5027859	0.0449		

group2	ch_dist6min_m60 LSMEAN	95% Confidence Limits	
**Home-based exercise**	36.730321	14.007520	59.453121
**Control**	-23.254583	-45.971452	-0.537713

Least Squares Means for Effect group2				
i	j	Difference Between Means	95% Confidence Limits for LSMean(i)-LSMean(j)	
**1**	**2**	59.984903	39.294635	80.675172

```
**LITE;
```

Group mean and stddev

The MEANS Procedure

Analysis Variable : ch_dist6min_m60				
group2	N Obs	N	Mean	Std Dev
**Control**	**59**	45	-10.91	49.59
**Home-based exercise**	**106**	85	34.55	74.98

Unadjusted

The GLM Procedure
Least Squares Means

group2	ch_dist6min_m60 LSMEAN	Standard Error	H0:LSMEAN=0	H0:LSMean1=LSMean2	
			Pr > |t|	t Value	Pr > |t|
**Home-based exercise**	34.5481835	7.3040795	<.0001	3.66	0.0004
**Control**	-10.9050667	10.0384971	0.2794		

© 2023 Thangada ND et al. *JAMA Network Open*.

group2	ch_dist6min_m60 LSMEAN	95% Confidence Limits	
**Home-based exercise**	34.548184	20.095814	49.000553
**Control**	-10.905067	-30.767948	8.957815

Least Squares Means for Effect group2				
i	j	Difference Between Means	95% Confidence Limits for LSMean(i)-LSMean(j)	
**1**	**2**	45.453250	20.888946	70.017554

Adjusted IPD

The GLM Procedure
Least Squares Means

group2	ch_dist6min_m60 LSMEAN	Standard Error	H0:LSMEAN=0	H0:LSMean1=LSMean2	
			Pr > |t|	t Value	Pr > |t|
**Home-based exercise**	43.7667964	11.9090504	0.0004	4.00	0.0001
**Control**	-7.6387725	14.5772830	0.6012		

group2	ch_dist6min_m60 LSMEAN	95% Confidence Limits	
**Home-based exercise**	43.766796	20.189690	67.343903
**Control**	-7.638772	-36.498349	21.220804

Least Squares Means for Effect group2				
i	j	Difference Between Means	95% Confidence Limits for LSMean(i)-LSMean(j)	
**1**	**2**	51.405569	25.956550	76.854588

```
**Home-based exercise studies combined;
```

Unadjusted

The GLM Procedure
Least Squares Means

group2	ch_dist6min_m60 LSMEAN	Standard Error	H0:LSMEAN=0	H0:LSMean1=LSMean2	
			Pr > |t|	t Value	Pr > |t|
**Home-based exercise**	38.6264196	5.2881357	<.0001	6.25	<.0001

group2	ch_dist6min_m60 LSMEAN	Standard Error	H0:LSMEAN=0	H0:LSMean1=LSMean2	
			Pr > |t|	t Value	Pr > |t|
**Control**	-12.1103745	6.1574025	0.0501		

group2	ch_dist6min_m60 LSMEAN	95% Confidence Limits	
**Home-based exercise**	38.626420	28.219312	49.033527
**Control**	-12.110374	-24.228209	0.007460

Least Squares Means for Effect group2				
i	j	Difference Between Means	95% Confidence Limits for LSMean(i)-LSMean(j)	
**1**	**2**	50.736794	34.756110	66.717479

Adjusted IPD

The GLM Procedure
Least Squares Means

group2	ch_dist6min_m60 LSMEAN	Standard Error	H0:LSMEAN=0	H0:LSMean1=LSMean2	
			Pr > |t|	t Value	Pr > |t|
**Home-based exercise**	38.7115598	8.0983393	<.0001	6.90	<.0001
**Control**	-16.8832812	8.8723027	0.0580		

group2	ch_dist6min_m60 LSMEAN	95% Confidence Limits	
**Home-based exercise**	38.711560	22.772356	54.650763
**Control**	-16.883281	-34.345805	0.579242

Least Squares Means for Effect group2				
i	j	Difference Between Means	95% Confidence Limits for LSMean(i)-LSMean(j)	
**1**	**2**	55.594841	39.731767	71.457915

```
**maximal treadmill walking distance;

**GOALS;
```

Group mean and stddev

© 2023 Thangada ND et al. *JAMA Network Open*.

The MEANS Procedure

Analysis Variable : ch_t_dist_meter60				
**group2**	**N Obs**	**N**	**Mean**	**Std Dev**
**Control**	**92**	82	33.03	161.61
**Home-based exercise**	**92**	82	89.26	173.40

Unadjusted

The GLM Procedure
Least Squares Means

group2	ch_t_dist_meter60 LSMEAN	Standard Error	H0:LSMEAN=0	H0:LSMean1=LSMean2	
			Pr > |t|	t Value	Pr > |t|
**Home-based exercise**	89.2553522	18.5093785	<.0001	2.15	0.0332
**Control**	33.0291688	18.5093785	0.0762		

group2	ch_t_dist_meter60 LSMEAN	95% Confidence Limits	
**Home-based exercise**	89.255352	52.704590	125.806114
**Control**	33.029169	-3.521593	69.579931

Least Squares Means for Effect group2				
**i**	**j**	**Difference Between Means**	**95% Confidence Limits for LSMean(i)-LSMean(j)**	
**1**	**2**	56.226183	4.535600	107.916767

Adjusted IPD

The GLM Procedure
Least Squares Means

group2	ch_t_dist_meter60 LSMEAN	Standard Error	H0:LSMEAN=0	H0:LSMean1=LSMean2	
			Pr > |t|	t Value	Pr > |t|
**Home-based exercise**	62.1937119	29.3394979	0.0356	1.97	0.0512
**Control**	9.8297283	30.0665101	0.7442		

group2	ch_t_dist_meter60 LSMEAN	95% Confidence Limits	
**Home-based exercise**	62.193712	4.236845	120.150578
**Control**	9.829728	-49.563269	69.222725

© 2023 Thangada ND et al. *JAMA Network Open*.

Least Squares Means for Effect group2				
**i**	**j**	**Difference Between Means**	**95% Confidence Limits for LSMean(i)-LSMean(j)**	
**1**	**2**	52.363984	-0.266896	104.994863

```
**LITE;
```

Group mean and stddev

The MEANS Procedure

Analysis Variable : ch_t_dist_meter60				
**group2**	**N Obs**	**N**	**Mean**	**Std Dev**
**Control**	**59**	31	38.60	120.76
**Home-based exercise**	**106**	59	97.78	161.23

Unadjusted

The GLM Procedure
Least Squares Means

group2	ch_t_dist_meter60 LSMEAN	Standard Error	H0:LSMEAN=0	H0:LSMean1=LSMean2	
			**Pr > |t|**	**t Value**	**Pr > |t|**
**Home-based exercise**	97.7767403	19.3559590	<.0001	1.79	0.0762
**Control**	38.5968568	26.7029875	0.1519		

group2	ch_t_dist_meter60 LSMEAN	95% Confidence Limits	
**Home-based exercise**	97.776740	59.310839	136.242641
**Control**	38.596857	-14.469720	91.663433

Least Squares Means for Effect group2				
**i**	**j**	**Difference Between Means**	**95% Confidence Limits for LSMean(i)-LSMean(j)**	
**1**	**2**	59.179884	-6.361608	124.721375

Adjusted IPD

The GLM Procedure

Least Squares Means

group2	ch_t_dist_meter60 LSMEAN	Standard Error	H0:LSMEAN=0	H0:LSMean1=LSMean2	
			Pr > |t|	t Value	Pr > |t|
**Home-based exercise**	118.076122	32.034438	0.0004	1.53	0.1297
**Control**	65.389149	40.619474	0.1113		

group2	ch_t_dist_meter60 LSMEAN	95% Confidence Limits	
**Home-based exercise**	118.076122	54.337639	181.814604
**Control**	65.389149	-15.430863	146.209161

Least Squares Means for Effect group2				
i	j	Difference Between Means	95% Confidence Limits for LSMean(i)-LSMean(j)	
**1**	**2**	52.686973	-15.798772	121.172717

```
**Home-based exercise studies combined;
```

Unadjusted

The GLM Procedure
Least Squares Means

group2	ch_t_dist_meter60 LSMEAN	Standard Error	H0:LSMEAN=0	H0:LSMean1=LSMean2	
			Pr > |t|	t Value	Pr > |t|
**Home-based exercise**	93.4206491	13.6598277	<.0001	2.78	0.0058
**Control**	36.2156076	15.8822383	0.0234		

group2	ch_t_dist_meter60 LSMEAN	95% Confidence Limits	
**Home-based exercise**	93.420649	66.518162	120.323136
**Control**	36.215608	4.936171	67.495044

Least Squares Means for Effect group2				
i	j	Difference Between Means	95% Confidence Limits for LSMean(i)-LSMean(j)	
**1**	**2**	57.205041	16.744002	97.666081

Adjusted IPD

The GLM Procedure
Least Squares Means

group2	ch_t_dist_meter60 LSMEAN	Standard Error	H0:LSMEAN=0	H0:LSMean1=LSMean2	
			Pr > |t|	t Value	Pr > |t|
**Home-based exercise**	82.1526162	21.3455198	0.0002	2.58	0.0105
**Control**	28.5043233	23.8416995	0.2330		

group2	ch_t_dist_meter60 LSMEAN	95% Confidence Limits	
**Home-based exercise**	82.152616	40.107620	124.197612
**Control**	28.504323	-18.457483	75.466129

Least Squares Means for Effect group2				
i	j	Difference Between Means	95% Confidence Limits for LSMean(i)-LSMean(j)	
**1**	**2**	53.648293	12.654434	94.642152

```
**pain-free treadmill walking distance;

**GOALS;
```

Group mean and stddev

The MEANS Procedure

Analysis Variable : ch_on_dist_meter60				
group2	N Obs	N	Mean	Std Dev
**Control**	**92**	82	25.30	124.61
**Home-based exercise**	**92**	82	75.28	156.64

Unadjusted

The GLM Procedure
Least Squares Means

group2	ch_on_dist_meter60 LSMEAN	Standard Error	H0:LSMEAN=0	H0:LSMean1=LSMean2	
			Pr > |t|	t Value	Pr > |t|
**Home-based exercise**	75.2771746	15.6298268	<.0001	2.26	0.0251
**Control**	25.2986478	15.6298268	0.1075		

group2	ch_on_dist_meter60 LSMEAN	95% Confidence Limits	
**Home-based exercise**	75.277175	44.412709	106.141640
**Control**	25.298648	-5.565818	56.163113

Least Squares Means for Effect group2				
i	j	Difference Between Means	95% Confidence Limits for LSMean(i)-LSMean(j)	
**1**	**2**	49.978527	6.329581	93.627473

Adjusted IPD

The GLM Procedure
Least Squares Means

group2	ch_on_dist_meter60 LSMEAN	Standard Error	H0:LSMEAN=0	H0:LSMean1=LSMean2	
			Pr > |t|	t Value	Pr > |t|
**Home-based exercise**	63.4101537	24.3113861	0.0100	1.94	0.0546
**Control**	20.6597010	24.6077372	0.4024		

group2	ch_on_dist_meter60 LSMEAN	95% Confidence Limits	
**Home-based exercise**	63.410154	15.385754	111.434553
**Control**	20.659701	-27.950106	69.269508

Least Squares Means for Effect group2				
i	j	Difference Between Means	95% Confidence Limits for LSMean(i)-LSMean(j)	
**1**	**2**	42.750453	-0.862145	86.363051

```
**LITE;
```

Group mean and stddev

The MEANS Procedure

Analysis Variable : ch_on_dist_meter60				
**group2**	**N Obs**	**N**	**Mean**	**Std Dev**
**Control**	**59**	31	62.92	185.26
**Home-based exercise**	**106**	59	92.36	236.10

Unadjusted

The GLM Procedure
Least Squares Means

group2	ch_on_dist_meter60 LSMEAN	Standard Error	H0:LSMEAN=0	H0:LSMean1=LSMean2	
			Pr > |t|	t Value	Pr > |t|
**Home-based exercise**	92.3630102	28.6537229	0.0018	0.60	0.5481
**Control**	62.9244852	39.5299455	0.1150		

group2	ch_on_dist_meter60 LSMEAN	95% Confidence Limits	
**Home-based exercise**	92.363010	35.419757	149.306263
**Control**	62.924485	-15.632975	141.481945

Least Squares Means for Effect group2				
**i**	**j**	**Difference Between Means**	**95% Confidence Limits for LSMean(i)-LSMean(j)**	
**1**	**2**	29.438525	-67.586258	126.463308

Adjusted IPD

The GLM Procedure
Least Squares Means

group2	ch_on_dist_meter60 LSMEAN	Standard Error	H0:LSMEAN=0	H0:LSMean1=LSMean2	
			Pr > |t|	t Value	Pr > |t|
**Home-based exercise**	99.4678951	43.4179521	0.0246	1.18	0.2400
**Control**	44.5476452	54.9033027	0.4195		

group2	ch_on_dist_meter60 LSMEAN	95% Confidence Limits	
**Home-based exercise**	99.467895	13.079790	185.856001
**Control**	44.547645	-64.692705	153.787996

Least Squares Means for Effect group2				
i	j	Difference Between Means	95% Confidence Limits for LSMean(i)-LSMean(j)	
**1**	**2**	54.920250	-37.392982	147.233482

```
**Home-based exercise studies combined;
```

Unadjusted

The GLM Procedure
Least Squares Means

group2	ch_on_dist_meter60 LSMEAN	Standard Error	H0:LSMEAN=0	H0:LSMean1=LSMean2	
			Pr > |t|	t Value	Pr > |t|
**Home-based exercise**	84.4834835	14.6905162	<.0001	1.95	0.0518
**Control**	41.3119259	17.0806166	0.0163		

group2	ch_on_dist_meter60 LSMEAN	95% Confidence Limits	
**Home-based exercise**	84.483483	55.551096	113.415871
**Control**	41.311926	7.672331	74.951521

Least Squares Means for Effect group2				
i	j	Difference Between Means	95% Confidence Limits for LSMean(i)-LSMean(j)	
**1**	**2**	43.171558	-0.342429	86.685544

Adjusted IPD

The GLM Procedure
Least Squares Means

group2	ch_on_dist_meter60 LSMEAN	Standard Error	H0:LSMEAN=0	H0:LSMean1=LSMean2	
			Pr > |t|	t Value	Pr > |t|
**Home-based exercise**	73.1889135	22.1043704	0.0011	1.96	0.0515
**Control**	31.1726401	24.4442777	0.2034		

group2	ch_on_dist_meter60 LSMEAN	95% Confidence Limits	
**Home-based exercise**	73.188914	29.649184	116.728643
**Control**	31.172640	-16.976085	79.321365

Least Squares Means for Effect group2				
**i**	**j**	**Difference Between Means**	**95% Confidence Limits for LSMean(i)-LSMean(j)**	
**1**	**2**	42.016273	-0.282796	84.315343

```
**WIQ distance score;

**GOALS;
```

Group mean and stddev

The MEANS Procedure

Analysis Variable : ch_distscr60				
**group2**	**N Obs**	**N**	**Mean**	**Std Dev**
**Control**	**92**	83	1.45	20.00
**Home-based exercise**	**92**	84	11.61	26.94

Unadjusted

The GLM Procedure
Least Squares Means

group2	ch_distscr60 LSMEAN	Standard Error	H0:LSMEAN=0	H0:LSMean1=LSMean2	
			**Pr > |t|**	**t Value**	**Pr > |t|**
**Home-based exercise**	11.6105249	2.5910058	<.0001	2.77	0.0063
**Control**	1.4469811	2.6065676	0.5796		

group2	ch_distscr60 LSMEAN	95% Confidence Limits	
**Home-based exercise**	11.610525	6.494725	16.726325
**Control**	1.446981	-3.699545	6.593507

Least Squares Means for Effect group2				
**i**	**j**	**Difference Between Means**	**95% Confidence Limits for LSMean(i)-LSMean(j)**	
**1**	**2**	10.163544	2.906951	17.420137

Adjusted IPD

The GLM Procedure
Least Squares Means

group2	ch_distscr60 LSMEAN	Standard Error	H0:LSMEAN=0	H0:LSMean1=LSMean2	
			Pr > |t|	t Value	Pr > |t|
**Home-based exercise**	8.70143523	3.82790139	0.0244	3.24	0.0015
**Control**	-2.45904709	3.94482852	0.5339		

group2	ch_distscr60 LSMEAN	95% Confidence Limits	
**Home-based exercise**	8.701435	1.140978	16.261893
**Control**	-2.459047	-10.250447	5.332352

Least Squares Means for Effect group2				
i	j	Difference Between Means	95% Confidence Limits for LSMean(i)-LSMean(j)	
**1**	**2**	11.160482	4.354940	17.966024

```
**LITE;
```

Group mean and stddev

The MEANS Procedure

Analysis Variable : ch_distscr60				
group2	N Obs	N	Mean	Std Dev
**Control**	**59**	47	1.15	24.31
**Home-based exercise**	**106**	86	8.72	21.78

Unadjusted

The GLM Procedure
Least Squares Means

group2	ch_distscr60 LSMEAN	Standard Error	H0:LSMEAN=0	H0:LSMean1=LSMean2	
			Pr > |t|	t Value	Pr > |t|
**Home-based exercise**	8.71927854	2.44799136	0.0005	1.84	0.0683

group2	ch_distscr60 LSMEAN	Standard Error	H0:LSMEAN=0	H0:LSMean1=LSMean2	
			Pr > |t|	t Value	Pr > |t|
**Control**	1.14845261	3.31138882	0.7293		

group2	ch_distscr60 LSMEAN	95% Confidence Limits	
**Home-based exercise**	8.719279	3.876568	13.561989
**Control**	1.148453	-5.402264	7.699170

Least Squares Means for Effect group2				
i	j	Difference Between Means	95% Confidence Limits for LSMean(i)-LSMean(j)	
**1**	**2**	7.570826	-0.575568	15.717220

Adjusted IPD

The GLM Procedure
Least Squares Means

group2	ch_distscr60 LSMEAN	Standard Error	H0:LSMEAN=0	H0:LSMean1=LSMean2	
			Pr > |t|	t Value	Pr > |t|
**Home-based exercise**	6.41927519	3.84610103	0.0976	2.37	0.0195
**Control**	-3.41847803	4.66820352	0.4654		

group2	ch_distscr60 LSMEAN	95% Confidence Limits	
**Home-based exercise**	6.419275	-1.193236	14.031786
**Control**	-3.418478	-12.658160	5.821204

Least Squares Means for Effect group2				
i	j	Difference Between Means	95% Confidence Limits for LSMean(i)-LSMean(j)	
**1**	**2**	9.837753	1.611502	18.064005

```
**Home-based exercise studies combined;
```

Unadjusted

The GLM Procedure
Least Squares Means

group2	ch_distscr60 LSMEAN	Standard Error	H0:LSMEAN=0	H0:LSMean1=LSMean2	
			Pr > |t|	t Value	Pr > |t|
**Home-based exercise**	10.1585894	1.7839933	<.0001	3.31	0.0010
**Control**	1.0873078	2.0747585	0.6006		

group2	ch_distscr60 LSMEAN	95% Confidence Limits	
**Home-based exercise**	10.158589	6.647720	13.669459
**Control**	1.087308	-2.995783	5.170398

Least Squares Means for Effect group2				
i	j	Difference Between Means	95% Confidence Limits for LSMean(i)-LSMean(j)	
**1**	**2**	9.071282	3.681949	14.460614

Adjusted IPD

The GLM Procedure
Least Squares Means

group2	ch_distscr60 LSMEAN	Standard Error	H0:LSMEAN=0	H0:LSMean1=LSMean2	
			Pr > |t|	t Value	Pr > |t|
**Home-based exercise**	6.64007401	2.64137430	0.0125	4.01	<.0001
**Control**	-3.86389655	2.92318376	0.1873		

group2	ch_distscr60 LSMEAN	95% Confidence Limits	
**Home-based exercise**	6.640074	1.441380	11.838769
**Control**	-3.863897	-9.617242	1.889449

Least Squares Means for Effect group2				
i	j	Difference Between Means	95% Confidence Limits for LSMean(i)-LSMean(j)	
**1**	**2**	10.503971	5.353215	15.654726

```
**WIQ speed score;

**GOALS;
```

Group mean and stddev

The MEANS Procedure

Analysis Variable : ch_speedscr60				
**group2**	**N Obs**	**N**	**Mean**	**Std Dev**
**Control**	**92**	85	1.84	23.66
**Home-based exercise**	**92**	84	11.57	24.11

Unadjusted

The GLM Procedure
Least Squares Means

group2	ch_speedscr60 LSMEAN	Standard Error	H0:LSMEAN=0	H0:LSMean1=LSMean2	
			Pr > |t|	t Value	Pr > |t|
**Home-based exercise**	11.5683230	2.6062093	<.0001	2.65	0.0089
**Control**	1.8414322	2.5908333	0.4782		

group2	ch_speedscr60 LSMEAN	95% Confidence Limits	
**Home-based exercise**	11.568323	6.422960	16.713686
**Control**	1.841432	-3.273575	6.956439

Least Squares Means for Effect group2				
**i**	**j**	**Difference Between Means**	**95% Confidence Limits for LSMean(i)-LSMean(j)**	
**1**	**2**	9.726891	2.471681	16.982100

Adjusted IPD

The GLM Procedure
Least Squares Means

group2	ch_speedscr60 LSMEAN	Standard Error	H0:LSMEAN=0	H0:LSMean1=LSMean2	
			Pr > |t|	t Value	Pr > |t|
**Home-based exercise**	12.8474914	3.5586129	0.0004	2.65	0.0088
**Control**	4.1658763	3.5488608	0.2422		

group2	ch_speedscr60 LSMEAN	95% Confidence Limits	
Home-based exercise	12.847491	5.819581	19.875402
Control	4.165876	-2.842774	11.174527

Least Squares Means for Effect group2				
i	j	Difference Between Means	95% Confidence Limits for LSMean(i)-LSMean(j)	
1	2	8.681615	2.214677	15.148553

```
**LITE;
```

Group mean and stddev

The MEANS Procedure

Analysis Variable : ch_speedscr60				
group2	N Obs	N	Mean	Std Dev
Control	59	47	-4.95	23.42
Home-based exercise	106	86	7.13	20.42

Unadjusted

The GLM Procedure
Least Squares Means

group2	ch_speedscr60 LSMEAN	Standard Error	H0:LSMEAN=0	H0:LSMean1=LSMean2	
			Pr > |t|	t Value	Pr > |t|
Home-based exercise	7.12841254	2.32051351	0.0026	3.09	0.0024
Control	-4.94912118	3.13895001	0.1173		

group2	ch_speedscr60 LSMEAN	95% Confidence Limits	
Home-based exercise	7.128413	2.537883	11.718942
Control	-4.949121	-11.158713	1.260471

Least Squares Means for Effect group2				
i	j	Difference Between Means	95% Confidence Limits for LSMean(i)-LSMean(j)	
1	2	12.077534	4.355358	19.799709

Adjusted IPD

The GLM Procedure
Least Squares Means

group2	ch_speedscr60 LSMEAN	Standard Error	H0:LSMEAN=0	H0:LSMean1=LSMean2	
			Pr > |t|	t Value	Pr > |t|
Home-based exercise	6.12055864	3.36877113	0.0717	2.93	0.0041
Control	-4.39559401	4.04566568	0.2794		

group2	ch_speedscr60 LSMEAN	95% Confidence Limits	
Home-based exercise	6.120559	-0.547183	12.788300
Control	-4.395594	-12.403100	3.611912

Least Squares Means for Effect group2				
i	j	Difference Between Means	95% Confidence Limits for LSMean(i)-LSMean(j)	
1	2	10.516153	3.400595	17.631710

```
**Home-based exercise studies combined;
```

Unadjusted

The GLM Procedure
Least Squares Means

group2	ch_speedscr60 LSMEAN	Standard Error	H0:LSMEAN=0	H0:LSMean1=LSMean2	
			Pr > |t|	t Value	Pr > |t|
Home-based exercise	9.35411941	1.75219721	<.0001	4.00	<.0001
Control	-1.35623543	2.02542104	0.5036		

© 2023 Thangada ND et al. *JAMA Network Open*.

group2	ch_speedscr60 LSMEAN	95% Confidence Limits	
**Home-based exercise**	9.354119	5.905919	12.802320
**Control**	-1.356235	-5.342122	2.629651

Least Squares Means for Effect group2				
i	j	Difference Between Means	95% Confidence Limits for LSMean(i)-LSMean(j)	
**1**	**2**	10.710355	5.435469	15.985240

Adjusted IPD

The GLM Procedure
Least Squares Means

group2	ch_speedscr60 LSMEAN	Standard Error	H0:LSMEAN=0	H0:LSMean1=LSMean2	
			Pr > |t|	t Value	Pr > |t|
**Home-based exercise**	8.71994287	2.41113078	0.0004	4.02	<.0001
**Control**	-0.95164858	2.61629477	0.7163		

group2	ch_speedscr60 LSMEAN	95% Confidence Limits	
**Home-based exercise**	8.719943	3.974545	13.465341
**Control**	-0.951649	-6.100834	4.197537

Least Squares Means for Effect group2				
i	j	Difference Between Means	95% Confidence Limits for LSMean(i)-LSMean(j)	
**1**	**2**	9.671591	4.941003	14.402180

```
**WIQ stair-climbing score;

**GOALS;
```

Group mean and stddev

The MEANS Procedure

Analysis Variable : ch_climbscr60				
group2	N Obs	N	Mean	Std Dev
**Control**	**92**	85	0.10	31.17
**Home-based exercise**	**92**	84	8.73	22.29

Unadjusted

The GLM Procedure
Least Squares Means

group2	ch_climbscr60 LSMEAN	Standard Error	H0:LSMEAN=0	H0:LSMean1=LSMean2	
			Pr > |t|	t Value	Pr > |t|
**Home-based exercise**	8.73015873	2.95952041	0.0036	2.07	0.0401
**Control**	0.09803922	2.94205996	0.9735		

group2	ch_climbscr60 LSMEAN	95% Confidence Limits	
**Home-based exercise**	8.730159	2.887264	14.573054
**Control**	0.098039	-5.710384	5.906463

Least Squares Means for Effect group2				
i	j	Difference Between Means	95% Confidence Limits for LSMean(i)-LSMean(j)	
**1**	**2**	8.632120	0.393357	16.870882

Adjusted IPD

The GLM Procedure
Least Squares Means

group2	ch_climbscr60 LSMEAN	Standard Error	H0:LSMEAN=0	H0:LSMean1=LSMean2	
			Pr > |t|	t Value	Pr > |t|
**Home-based exercise**	11.9056087	3.8257412	0.0022	2.64	0.0090
**Control**	2.6049590	3.8152021	0.4957		

group2	ch_climbscr60 LSMEAN	95% Confidence Limits	
**Home-based exercise**	11.905609	4.350146	19.461071
**Control**	2.604959	-4.929690	10.139608

Least Squares Means for Effect group2				
i	j	Difference Between Means	95% Confidence Limits for LSMean(i)-LSMean(j)	
**1**	**2**	9.300650	2.356053	16.245246

```
**LITE;
```

Group mean and stddev

The MEANS Procedure

Analysis Variable : ch_climbscr60

group2	N Obs	N	Mean	Std Dev
**Control**	**59**	47	-4.96	28.71
**Home-based exercise**	**106**	86	4.41	22.99

Unadjusted

The GLM Procedure
Least Squares Means

group2	ch_climbscr60 LSMEAN	Standard Error	H0:LSMEAN=0	H0:LSMean1=LSMean2	
			Pr > |t|	t Value	Pr > |t|
**Home-based exercise**	4.40891473	2.71177380	0.1064	2.05	0.0419
**Control**	-4.96453901	3.66820634	0.1783		

group2	ch_climbscr60 LSMEAN	95% Confidence Limits	
**Home-based exercise**	4.408915	-0.955621	9.773450
**Control**	-4.964539	-12.221126	2.292048

Least Squares Means for Effect group2				
i	j	Difference Between Means	95% Confidence Limits for LSMean(i)-LSMean(j)	
**1**	**2**	9.373454	0.349248	18.397660

Adjusted IPD

The GLM Procedure
Least Squares Means

group2	ch_climbscr60 LSMEAN	Standard Error	H0:LSMEAN=0	H0:LSMean1=LSMean2	
			Pr > |t|	t Value	Pr > |t|
**Home-based exercise**	7.78791378	4.14176998	0.0624	2.82	0.0056
**Control**	-4.88240612	4.99945682	0.3307		

group2	ch_climbscr60 LSMEAN	95% Confidence Limits	
**Home-based exercise**	7.787914	-0.409809	15.985637

group2	ch_climbscr60 LSMEAN	95% Confidence Limits	
**Control**	-4.882406	-14.777732	5.012919

Least Squares Means for Effect group2				
**i**	**j**	**Difference Between Means**	**95% Confidence Limits for LSMean(i)-LSMean(j)**	
**1**	**2**	12.670320	3.783843	21.556797

```
**Home-based exercise studies combined;
```

Unadjusted

The GLM Procedure
Least Squares Means

group2	ch_climbscr60 LSMEAN	Standard Error	H0:LSMEAN=0	H0:LSMean1=LSMean2	
			**Pr > |t|**	**t Value**	**Pr > |t|**
**Home-based exercise**	6.57135066	2.01187320	0.0012	2.91	0.0039
**Control**	-2.37092893	2.32558885	0.3088		

group2	ch_climbscr60 LSMEAN	95% Confidence Limits	
**Home-based exercise**	6.571351	2.612126	10.530576
**Control**	-2.370929	-6.947524	2.205666

Least Squares Means for Effect group2				
**i**	**j**	**Difference Between Means**	**95% Confidence Limits for LSMean(i)-LSMean(j)**	
**1**	**2**	8.942280	2.885655	14.998904

Adjusted IPD

The GLM Procedure
Least Squares Means

group2	ch_climbscr60 LSMEAN	Standard Error	H0:LSMEAN=0	H0:LSMean1=LSMean2	
			**Pr > |t|**	**t Value**	**Pr > |t|**
**Home-based exercise**	8.95034743	2.76278943	0.0013	3.81	0.0002
**Control**	-1.60993892	3.01175406	0.5934		

group2	ch_climbscr60 LSMEAN	95% Confidence Limits	
**Home-based exercise**	8.950347	3.512842	14.387852
**Control**	-1.609939	-7.537437	4.317559

Least Squares Means for Effect group2				
i	j	Difference Between Means	95% Confidence Limits for LSMean(i)-LSMean(j)	
**1**	**2**	10.560286	5.106313	16.014260
