# Supplementary material for: Home-Based Walking Exercise and Supervised Treadmill Exercise in Patients With Peripheral Artery Disease: An Individual Participant Data Meta-Analysis
Source: JAMA Netw Open. 2023 Sep 21;6(9):e2334590. doi: 10.1001/jamanetworkopen.2023.34590 (PMC10514734; doi:10.1001/jamanetworkopen.2023.34590)
Supplement: Supplement 2. — Data Sharing Statement [file jamanetwopen-e2334590-s002.pdf]

Data Sharing Statement

Thangada. Home-Based Walking Exercise and Supervised Treadmill Exercise in Patients With Peripheral Artery Disease. *JAMA Netw Open*. Published September 19, 2023.
doi:10.1001/jamanetworkopen.2023.34590

Data
Data available: Yes
Data types: Other (please specify)
Additional Information: Summary data can be made available
How to access data: Mary M. McDermott, MD can be reached at mdm608@Northwestern.edu
When available: With publication

Supporting Documents
Document types: None

Additional Information
Who can access the data: Upon reasonable request to the study Principal Investigator
Types of analyses: For a specific purpose
Mechanisms of data availability: After approval of a proposal, without investigator support
Any additional restrictions: None
